# Supplementary material for: How does a poetry audiobook app improve the perception of well-being in older adults? A study protocol
Source: PLoS One. 2024 Oct 31;19(10):e0312463. doi: 10.1371/journal.pone.0312463 (PMC11527330; doi:10.1371/journal.pone.0312463)
Supplement: S1 Appendix — A questionnaire to obtain the older adults’ perception of Hedonic, Eudaimonic and Social well-being before and after the intervention. (PDF) [file pone.0312463.s002.pdf]

Well-being questionnaire in audiobook application experience for older adults

Id:______________________________________

Date:____________________________________

Instruction

The purpose of this brief questionnaire is to learn about your well-being in two stages, before and after using the audiobook mobile application.

It is important to consider the following definitions:

Technological resource will be understood as elements such as cell phones, computers, tablets, and mobile applications.

Mobile application will be understood as an application installed on the smartphone to perform a certain function. Example: communicate with other people: WhatsApp; food orders: Pedidos Ya; watch videos: YouTube.

For each question you must mark with an **X** to answer whether you "strongly disagree", "disagree", "neither agree nor disagree", "agree" or "strongly agree" with the statement.

Part A: Assessing well-being before the audiobook mobile application experience.

Question 1
Am I interested in learning how to use different technological resources?

Strongly disagree	Disagree	Neither agree nor disagree	Agree	Strongly agree

Question 2
Do I feel that I am interested in applying technological resources to my life?

Strongly disagree	Disagree	Neither agree nor disagree	Agree	Strongly agree

Question 3
Do I have a positive perception of the use of technological resources?

Strongly disagree	Disagree	Neither agree nor disagree	Agree	Strongly agree

Question 4
Do I feel that I have the same abilities as people my own age in the use of technological resources?

Strongly disagree	Disagree	Neither agree nor disagree	Agree	Strongly agree

Question 5
Am I satisfied with how I use technological resources?

Strongly disagree	Disagree	Neither agree nor disagree	Agree	Strongly agree

Question 6
Do I feel I have sufficient access to literature?

Strongly disagree	Disagree	Neither agree nor disagree	Agree	Strongly agree

Part B: Assessing well-being after the audiobook mobile application experience.

Question 1
Am I interested in learning how to use different technological resources?

Strongly disagree	Disagree	Neither agree nor disagree	Agree	Strongly agree

Question 2
Do I feel that I am interested in applying technological resources to my life?

Strongly disagree	Disagree	Neither agree nor disagree	Agree	Strongly agree

Question 3
Do I have a positive perception of the use of technological resources?

Strongly disagree	Disagree	Neither agree nor disagree	Agree	Strongly agree

Question 4
Do I feel that I have the same abilities as people my own age in the use of technological resources?

Strongly disagree	Disagree	Neither agree nor disagree	Agree	Strongly agree

Question 5
Am I satisfied with how I use technological resources?

Strongly disagree	Disagree	Neither agree nor disagree	Agree	Strongly agree

Question 6
Do I feel I have sufficient access to literature?

Strongly disagree	Disagree	Neither agree nor disagree	Agree	Strongly agree

Question 7
Am I satisfied with the audiobook experience?

Strongly disagree	Disagree	Neither agree nor disagree	Agree	Strongly agree

Question 8
Do I feel that the use of the audiobook was meaningful to me?

Strongly disagree	Disagree	Neither agree nor disagree	Agree	Strongly agree

Question 9
Do I feel like I enjoyed the audiobook experience?

Strongly disagree	Disagree	Neither agree nor disagree	Agree	Strongly agree

Question 10
After using the audiobook, am I interested in using other mobile applications?

Strongly disagree	Disagree	Neither agree nor disagree	Agree	Strongly agree

Question 11
Do I feel that the audiobook experience allowed me greater access to literature?

Strongly disagree	Disagree	Neither agree nor disagree	Agree	Strongly agree

Question 12
Do I feel that the audiobook experience makes it easier for me to have new topics of conversation?

Strongly disagree	Disagree	Neither agree nor disagree	Agree	Strongly agree

Thank you very much for your collaboration!
