# Supplementary material for: How does a poetry audiobook app improve the perception of well-being in older adults? A study protocol
Source: PLoS One. 2024 Oct 31;19(10):e0312463. doi: 10.1371/journal.pone.0312463 (PMC11527330; doi:10.1371/journal.pone.0312463)
Supplement: S1 File — (PDF) [file pone.0312463.s003.pdf]

Protocolo Proyecto de investigación

I. DATOS GENERALES

**Título del proyecto**	Uso de aplicación de audiolibro en adultos mayores pertenecientes al Centro Comunitario de Rehabilitación y el impacto en su bienestar.
**Investigador(es) Responsables**	Valeria Espejo Videla
**Co-investigador(es)**	Laura Aravena Canese<br>Pedro Rossel Cid
**Institución Patrocinante**	Universidad de Concepción y Universidad Católica de la Santísima Concepción
**Fuente de financiamiento o autofinanciado**	Autofinanciado

SERVICIO DE SALUD CONCEPCIÓN
COMITÉ ÉTICO CIENTÍFICO
04 MAYO 2021
CONCEPCIÓN

II. DESCRIPCIÓN DEL PROYECTO

1. Justificación de la Propuesta y Estado del Arte

1.1 Adulto Mayor, Salud y Bienestar

La población de adultos mayores ha ido en aumento a nivel mundial, por lo que se han convertido en receptores directos de políticas públicas que favorecen su bienestar desde todos los ejes de su vida, tanto social, como de salud, tecnológico y cultural, entre otros. Las inequidades en cualquiera de estos ámbitos no debieran seguir aumentando si lo que se desea es mejorar su calidad de vida. Éste es el caso del uso de tecnologías y cómo a través de ellas se favorece el envejecimiento activo y el bienestar global de las personas mayores, o como en su ausencia se producen brechas aún mayores en las posibilidades de disfrutar la vida (Matas-Terrón et al., 2016).

Durante el proceso de la adultez mayor es importante mantener el interés y la participación activa en las oportunidades socioculturales que se puedan presentar. El objetivo de esta permanente actividad, continua y cotidiana, es prevenir el aislamiento social y familiar, y mantener la independencia (Villafuerte et al., 2017). Actividades comunes como escuchar la radio, música, o un audiolibro permiten el disfrute, el entretenimiento, la estimulación cognitiva y favorecen la relajación y el olvido de las preocupaciones (Forsblom et al., 2010).

SERVICIO DE SALUD CONCEPCIÓN
COMITÉ ÉTICO CIENTÍFICO
0 4 MAYO 2021
CONCEPCIÓN

Actualmente, la salud tiene una mirada integral y ya en 1948 la Organización Mundial de la Salud la define como “el completo bienestar físico, mental y social, y no solamente la ausencia de afecciones o enfermedades”. Esta visión es relevante en los adultos mayores ya que no solo considera lo biomédico, sino también los factores (Gallardo-Peralta & Sánchez-Moreno, 2019; Khosravi et al., 2016).

Una de las metas del trabajo en salud es buscar el bienestar y mejorar la calidad de vida de las personas mayores. La Organización Mundial de la Salud (OMS) señala que las políticas sanitarias deben orientarse a promover un envejecimiento saludable, entendido como “el proceso de fomentar y mantener la capacidad funcional que permite a la persona el bienestar en la vejez” (Organización Mundial de la Salud (OMS), 2006).

En el envejecimiento se aprecian cambios físicos, sociales y cognitivos, siendo un estado fisiológico y no un sinónimo de enfermedad. Por consiguiente, el envejecimiento es un estado biocultural relacionado a ámbitos temporales, sociales y ambientales (Martínez Fuentes & Fernández Díaz, 2008). Esta etapa del ciclo vital, tiende a asociarse a un proceso de pérdidas y a un periodo de crisis, lo que genera que algunos adultos mayores tengan dificultades para adaptarse a esta fase y por lo mismo se vuelvan más vulnerables, es así que es de gran relevancia que se generen acciones integrales en salud para apoyar a las personas mayores y lograr así un desarrollo permanente orientado a mejorar su bienestar, que puedan adoptar nuevos roles en diversos contextos y potenciar el crecimiento psicosocial y espiritual (Espinoza L., 2016).

Es así, que el bienestar es parte de la salud en su sentido más general y se manifiesta en todos los ámbitos de la actividad humana. Cuando un individuo se siente bien es más productivo, sociable, creativo, posee una proyección de futuro positiva, infunde felicidad y ésta implica capacidad de amar, trabajar, relacionarse socialmente y controlar el medio. Está demostrada la asociación entre algunos estados emocionales y las respuestas de enfrentamiento al estrés de un tipo u otro (García & González, 2000) citado por Abello et al., (2008).

Según Diener, 1994 citado en Zubieta & Delfino, 2010 el bienestar se puede caracterizar a partir de tres elementos: su carácter subjetivo, que descansa sobre la propia experiencia de la persona; su dimensión global que incluye la valoración del sujeto en todas las áreas de su vida; y la apreciación positiva, ya que su naturaleza va más allá de la mera ausencia de factores negativos.

Ryan y Deci (2001) citado en Ortiz Arriagada & Castro Salas, 2009 han propuesto una organización de los diferentes estudios del bienestar en dos grandes tradiciones, una relacionada fundamentalmente con la felicidad, la tradición hedónica, y otra ligada al desarrollo del potencial humano y a la realización de aquello que se puede ser, definida como la tradición eudaimónica.

El bienestar hedónico o subjetivo se entiende como “la categoría que utiliza una persona para juzgar de un modo general o global su vida” (Diener & Suh, 2000) argumentan que el Bienestar Subjetivo está integrado por las siguientes 3 dimensiones: la Satisfacción con la Vida, Afecto Positivo y Afecto Negativo.

Carol Ryff se refiere al bienestar eudaimónico o psicológico como el esfuerzo por perfeccionar el propio potencial, es el modo en que la vida adquiera un significado para uno mismo, con ciertos esfuerzos de superación y conseguir metas valiosas; la tarea central de las personas en su vida es reconocer y realizar al máximo todos sus talentos (Keyes et al., 2002). Para explicar el bienestar psicológico, Ryff formula un modelo multidimensional del desarrollo personal donde especifica seis dimensiones: autoaceptación, relaciones positivas

SERVICIO ... COMITÉ ÉTICO CIENTÍFICO 04 MAYO 2021 CONCEPCIÓN

con otras personas, autonomía, dominio del entorno, propósito en la vida, y crecimiento personal (Rodríguez, Negrón, Maldonado, Quiñones & Osorio, 2015) .

El bienestar social es la valoración que se hace de las circunstancias y el funcionamiento dentro de la sociedad Keyes, 1998, citado por (Blanco & Díaz, 2005). Está compuesto por las siguientes dimensiones: integración social, aceptación social, contribución social, actualización social, y coherencia social.

1.2 Adulto mayor y uso de tecnología

Se debe potenciar en los adultos mayores una aproximación positiva hacia la tecnológica, promoviendo el interés en la alfabetización digital que permita fortalecer el uso de los distintos recursos digitales, poniendo el foco en disminuir el temor al uso, y potenciando las habilidades en base a sus intereses y gustos (Matas-Terrón et al., 2016a). Desde esta perspectiva, el acceso y disponibilidad de literatura a través de tecnologías digitales podría ser un factor de mejora en el bienestar de las personas mayores.

La población adulta mayor se encuentra alejada del uso de las tecnologías de la información y en particular de las aplicaciones móviles, debido a diversas barreras, como por ejemplo, barreras físicas, de aceptación y de diseño (Fletcher & Jensen, 2016). Esta brecha en el uso de tecnologías digitales les priva de utilizar servicios que podrían impactar positivamente en su salud, tales como contenidos culturales, libros, cuentos o poemas que están accesibles en plataformas virtuales.

Las aplicaciones móviles, basadas en e-Health son recursos tecnológicos que funcionan como herramientas de información y entrega de soporte para promover la salud, prevenir enfermedades, monitorear diagnósticos y tratamientos, así como también ayudar a controlar los estilos de vida de los distintos usuarios (Vukovic et al., 2018).

El uso de las tecnologías de la información y comunicación (TIC) genera beneficios en la calidad de vida de las personas mayores e impactan en distintos ámbitos, entre ellos en el aprendizaje, la comunicación, las actividades diarias, el entretenimiento, los hobbies y la salud (Casamayou & Morales González, 2018). El estudio de Kim et al., 2017 examinó el grado en que las TIC mejoran el bienestar psicológico de los adultos mayores al facilitar su conexión social. Además, favorecer instancias que promuevan la salud a través de aplicaciones usables para el adulto mayor permitiría mejorar la percepción de la calidad de vida y aumentar la felicidad de la persona. El efecto de mejora encontrado fue moderado por la edad y por el estado de fragilidad y se observó que reduce el aislamiento social en esta población (Fang et al., 2018).

Una aplicación que permite conectarse con la literatura es el audiolibro, corresponde a la grabación de una obra literaria leída o dramatizada por una o varias personas en la cual se puede incorporar música o efectos (García-Rodríguez & Gómez-Díaz, 2019). En un inicio estuvo orientado a personas en situación de discapacidad visual o para personas que buscaban aprender otro idioma, actualmente se encuentra dirigido a toda la población en todo su ciclo vital (García-Rodríguez & Gómez-Díaz, 2019). Los audiolibros son un recurso que podría alcanzar una mayor cantidad de población adulta mayor, pudiendo superar barreras como el analfabetismo, dificultades del envejecimiento como la presbicia y alteraciones motoras entre otras (Ameri et al., 2017). Los audiolibros generan una experiencia positiva y puede mejorar el sentido de la vida incluyendo aspectos tanto del bienestar físico como mental (Ferrari, 2012).

Si bien ha habido investigación relacionada con libros electrónicos y libros de audio, ésta ha sido mayoritariamente en población de niños y adolescentes (Larson, 2015; Moyer,

SERVICIO DE SALUD CONCEPCION
COMITÉ ÉTICO CIENTÍFICO
0 4 MAYO 202[illegible]
CONCEPCIÓN

2012). De acuerdo a lo anterior y a la revisión realizada, hay una oportunidad de investigación y de realizar un aporte en la población de adultos mayores, considerando la utilización de audios de poemas para que ellos puedan escucharlos, puesto que casi no existe investigación relacionada y se ha visto reportado que para los adultos mayores puede ser una herramienta de utilidad (Macik et al., 2017; Smallfield & Kaldenberg, 2020).

Alstergren et al., desarrollaron "DreamScape", la que es una aplicación que permite crear, compartir y escuchar historias. Fue desarrollada para ser ejecutada tanto en un navegador como en dispositivos móviles. La idea principal de esta aplicación es que los usuarios pueden crear historias seleccionando distintas opciones a medida que la historia avanza (opción múltiple), historias que eventualmente pueden tener diferentes finales. Esta aplicación fue evaluada con 4 personas, cuyas edades estaban entre 22 y 60 años. Cabe mencionar que esta aplicación está más centrada en la creación de historias, como elemento de juego que le permita a las personas mejorar su bienestar. El elemento que permite escuchar la historia es complementario a lo anterior.

Endrstova et al., desarrollaron una unidad física para la reproducción de contenido de audio adaptado a necesidades y preferencias específicas de adultos mayores con discapacidad visual. En particular, esta unidad fue desarrollada pensando en metáforas conocidas para los usuarios, tales como encender y apagar la lectura abriendo y cerrando el libro, correspondientemente. También posee funciones tales como control de volumen, omitir capítulos, rebobinar, marcar como favorito y cambiar entre títulos de libros particulares que pueden ser presentados por el dispositivo. Se hicieron diversas evaluaciones, una de ellas con 7 adultos mayores con discapacidad visual de una edad promedio de 73 años, que vivían en una residencia para adultos mayores. La evaluación tuvo una duración de media hora.

Poerio & Totterdell, realizaron un ensayo controlado aleatorizado longitudinal, donde se estudió el efecto de los audiolibros sobre el bienestar de los adultos mayores. Los participantes fueron 94 adultos mayores que usaron durante un período de seis semanas un reproductor de MP3. Éste contenía los libros a ser escuchados. El estudio mostró que los sujetos que refirieron una mayor absorción y valoración del audiolibro también manifestaron un mayor bienestar postintervención, lo que se extendió hasta después de la finalización del libro. De acuerdo a los autores, el uso de un audiolibro puede tener un impacto positivo duradero en varios aspectos del bienestar de los adultos mayores.

Por otra parte, es importante que las herramientas orientadas a e-Health sean interactivas, interoperables, fáciles de usar, motivadoras, adaptables y accesible para distintos receptores (Kreps & Neuhauser, 2010). De este modo, conocer la usabilidad de una aplicación es fundamental para poder determinar si es eficiente y práctica en su uso.

Se entiende por usabilidad a la medida en que un sistema, producto o servicio puede ser utilizado por usuarios para lograr objetivos específicos con efectividad, eficiencia y satisfacción en un contexto de uso determinado (ISO 9241-11, 2018). De acuerdo con Rubin & Chisnell, para que un producto pueda ser usable (utilizable), éste debe ser útil, eficiente, efectivo, satisfactorio, fácil de aprender y accesible. Los mismos autores indican a que se refiere cada una de estas propiedades:

- Utilidad: El grado en que un producto le permite al usuario alcanzar sus objetivos, y es una evaluación de la disposición del usuario a usar el producto.
- Eficiencia: La rapidez con la que el objetivo del usuario se puede lograr de manera precisa y completa, generalmente es una medida de tiempo.

- Efectividad: La medida en que el producto se comporta de la manera que los usuarios esperan y la facilidad con que los usuarios pueden usarlo para hacer lo que desean. Esto generalmente se mide cuantitativamente con la tasa de error.
- Satisfacción: Las percepciones, los sentimientos y las opiniones del usuario sobre el producto, generalmente capturados a través de preguntas escritas y orales.
- Facilidad de Aprendizaje: Es parte de la efectividad y tiene que ver con la capacidad del usuario para operar el sistema a un nivel definido de competencia después de una cantidad predeterminada y un período de capacitación (que puede no tener tiempo). También puede referirse a la capacidad de usuarios poco frecuentes para volver a aprender el sistema después de períodos de inactividad.
- Accesibilidad: En un sentido amplio, la accesibilidad se trata de tener acceso a los productos necesarios para lograr un objetivo. En un sentido más reducido, lo que hace que los productos puedan ser utilizados por personas en situación de discapacidad.

Evaluar la usabilidad es una de las tareas más importantes a la hora de construir una interfaz de usuario de la herramienta de software, la que se convertirá en la parte visible de la interacción con la aplicación y probablemente con la que se producirán errores en dicha interacción. La idea en una evaluación es conseguir retroalimentación de parte de los usuarios, de tal forma de identificar los objetivos cumplidos o monitorizar el uso del producto. Por lo tanto, se debe tener en cuenta distintas características que puedan afectar el normal funcionamiento de la aplicación como la interactividad, la facilidad y por sobre todo la estética para acceder a ella (Zhang & Adipat, 2005).

1.3 Presentación del problema

A través del siguiente estudio se desea conocer el impacto de una aplicación móvil de audiolibro en el bienestar de las personas adultas mayores y la usabilidad de la aplicación móvil diseñada para esta población.

SERVICIO DE SALUD CONCEPCIÓN
COMITÉ ÉTICO CIENTÍFICO
04 MAYO 202[illegible]

1.3.1 Justificación del problema

En Chile en el último tiempo, se ha percibido un aumento acelerado de la población mayor y constituyen el 16,2% de la población general (Albala, 2020). Las personas mayores, deben adaptarse a los cambios que conlleva el envejecimiento, lo cual la hace vulnerable desde distintos aspectos, tanto económico, de acceso a la tecnología, de educación y en salud.

En el ámbito de salud, se pueden evidenciar declives tanto a nivel visual, cognitivo, como funcional; en relación los déficits visuales tienden a interferir con las actividades de la vida diaria y en el plano psicológico generando pérdida de la autoestima, aislamiento social, entre otros (Loh & Ogle, 2004). Con respecto a la funcionalidad en esta población, se observa la dificultad para realizar las actividades cotidianas impactando en actividades básicas o instrumentales (Mora Quezada et al., 2017).

Se ha estudiado el rol de la lectura en población mayor y se ha determinado que la lectura placentera se asocia a beneficios en la salud y el bienestar (Poerio & Totterdell, 2020). Otra alternativa que tienen los adultos mayores es la escucha de audiolibros que tiene un efecto importante en el bienestar y en el sentido de la vida (Poerio & Totterdell, 2020).

Debido a que existe una masificación en el uso de las tecnologías, hay un aumento de los consumidores digitales y nos encontramos en una cultura digital (Guaña-Moya et al., 2017), por lo que se torna relevante empoderar a la población mayor para que tengan un

mayor acceso a instancias tecnológicas gratuitas que les permita conectar con ámbitos tan potentes en un individuo inserto en comunidad, como son la literatura y cultura.

1.3.2 Impacto

La relevancia de determinar si el uso de audiolibro impacta en el bienestar de las personas mayores, es que se encuentra ligado a un beneficio en la salud desde un concepto biopsicosocial. Las personas mayores se encuentran aisladas del uso de la tecnología, por lo mismo se deben crear o adaptar herramientas tecnológicas a esta población en particular, ya que presentan necesidades y limitaciones puntuales y así lograr potenciar la facilidad en el uso de aplicaciones móviles y satisfacción con ellas u otros sistemas digitales. Al diseñar plataformas digitales específicamente orientadas a las personas mayores ellas son consideradas como miembros de la sociedad, y a la vez se propicia la disminución de las brechas tecnológicas de esta población y se motivan a ser parte de la comunidad digital.

1.3.3 Novedad

Al momento de la revisión bibliográfica no se encontraron estudios en Chile sobre el uso de audiolibros en población mayor, más aún que analicen el impacto que tiene en el bienestar, aunque sí existen estudios similares en otros países y para población de niños y adolescentes (Larson, 2015; Moyer, 2012; García-herrera et al., 2020; Ronquillo & Peña, 2017). El uso de la tecnología en los adultos mayores a través de una aplicación móvil de audiolibro busca potenciar una aproximación positiva hacia la tecnológica, promoviendo el interés en la alfabetización digital que permitan fortalecer el uso de los distintos recursos digitales poniendo el foco en disminuir el temor al uso y potenciando las habilidades en base a sus intereses y gustos (Matas-Terrón et al., 2016a).

La idea es que los adultos mayores integren la tecnología como recurso que les permita el acceso y disfrute de sus intereses, por ello se propone una aplicación móvil diseñada según sus características. Con lo anterior, abrir una puerta y acercarlos a espacios sociales como son el de acceder a la literatura y a la cultura.

Como ya fue comentado, los audiolibros generan una experiencia positiva y pueden mejorar el sentido de la vida incluyendo aspectos tanto del bienestar físico como mental (Poerio & Totterdell, 2020).

SERVICIO DE SALUD CONCEPCIÓN
COMITÉ ÉTICO CIENTÍFICO
04 MAYO 2021
CONCEPCIÓN

SERVICIO DE SALUD CONCEPCIÓN
COMITÉ ÉTICO CIENTÍFICO
0 4 MAYO 2021
CONCEPCIÓN

2. Objetivos/Hipótesis de Investigación

2.1 Pregunta de investigación

La escucha de audios de poemas utilizando una aplicación móvil ¿mejora la percepción del bienestar de los adultos mayores que se atienden en el Centro Comunitario de Rehabilitación (CCR) de Concepción?

2.2 Hipótesis

H1: El uso de una aplicación móvil que permite la escucha de audios de poemas produce un cambio positivo en la percepción del bienestar en adultos mayores.
H0: El uso de una aplicación móvil que permite la escucha de audios de poemas no produce un cambio positivo en la percepción del bienestar en adultos mayores.

2.3 Objetivos

2.3.1 Objetivo General

Determinar los cambios en la percepción del bienestar de los adultos mayores que se atienden en CCR Concepción, posterior al uso de una aplicación móvil para escuchar audios de poemas.

2.3.2 Objetivos Específicos

1. Modificar el lenguaje utilizado en el cuestionario de bienestar para que sea entendible y apropiado para adultos mayores.
2. Caracterizar a la población adulta mayor que se atiende en el CCR de Concepción.
3. Determinar los cambios en la percepción del bienestar hedónico en adultos mayores que se atienden en CCR antes y posterior al uso de la aplicación móvil de audiolibro.
4. Determinar los cambios en la percepción del bienestar eudaimónico de los adultos mayores que se atienden en CCR antes y posterior al uso de la aplicación móvil de audiolibro.
5. Determinar los cambios en la percepción del bienestar social de los adultos mayores que se atienden en CCR antes y posterior al uso de la aplicación móvil de audiolibro.

3. Material y Método

3.1 Metodología: Cualitativa/Cuantitativa/Mixta

Metodología del estudio cuantitativa, ya que nuestro propósito es responder a la pregunta de investigación, someter nuestra hipótesis a prueba y cumplir con los objetivos generales y específicos planteados (Hernández et al., 2014)

3.2 Diseño del estudio

El diseño de la investigación corresponde a un estudio cuasiexperimental (Hernández et al., 2014).
Es de tipo cuasiexperimental, ya que existe una intervención deliberada, es decir, los sujetos no se asignan al azar, y el grupo se encuentra conformado antes del experimento. El diseño corresponde a una preprueba/posprueba con un solo grupo; este es medido previo al uso del audiolibro, luego los adultos mayores usan la aplicación móvil por un mes y posteriormente se le aplica una evaluación final. Además, existe un punto de referencia inicial para ver qué nivel tenía el grupo en la variable dependiente antes del estímulo.

Es de tipo descriptivo ya que se buscará describir si existe un cambio en la percepción del bienestar de los adultos mayores pertenecientes al CCR Concepción, luego de utilizar una aplicación móvil de audiolibro. Las variables percepción de bienestar y usabilidad, serán medidas de manera cuantitativa para contrastar la hipótesis arriba planteada.

3.3 Variables

El estudio busca conocer las características demográficas, el bienestar de la persona pre y post uso de la aplicación móvil y la usabilidad de ésta. Las variables a considerar en este estudio son:

1. Características sociodemográficas
2. Bienestar hedónico
3. Bienestar eudaimónico
4. Bienestar social
5. Aplicación audiolibro para la escucha de poemas diseñado para el adulto mayor.

A continuación se detallan a nivel conceptual las variables

1. Las características sociodemográficas se entenderán como:
    a. Edad: Años cumplidos desde la fecha inscrita en el carnet de identidad.
    b. Sexo: Hombre o mujer de acuerdo a propia definición.
    c. Escolaridad: Años de escolaridad formal completados por el individuo.
    d. Competencias digitales

SERVICIO DE SALUD CONCEPCION
COMITÉ ÉTICO CIENTÍFICO
04 MAYO 2021
CONCEPCION

El bienestar se puede caracterizar a partir de tres ámbitos: bienestar hedónico, eudaimónico y social.

1. El bienestar hedónico o subjetivo se entiende como la categoría que utiliza una persona para juzgar de un modo general o global su vida" (Diener & Suh,2000).

2. El bienestar eudaimónico o psicológico como el esfuerzo por perfeccionar el propio potencial, es el modo en que la vida adquiera un significado para uno mismo, con ciertos esfuerzos de superación y conseguir metas valiosas; la tarea central de las personas en su vida es reconocer y realizar al máximo todos sus talentos (Romero Carrasco et al., 2007).

3. El bienestar social es la valoración que se hace de las circunstancias y el funcionamiento dentro de la sociedad (Blanco & Díaz, 2005)

4. Aplicación audiolibro para la escucha de poemas diseñado para el adulto mayor.

El audiolibro, corresponde a la grabación de una obra literaria leída o dramatizada por una o varias personas en la cual se puede incorporar música o efectos (García-Rodríguez & Gómez-Díaz, 2019). La aplicación audiolibro es una aplicación móvil, construida especialmente para este estudio. Funciona sobre el sistema operativo Android 5.0 o superior para teléfonos móviles. Está construida para adultos mayores, y se consideraron en su diseño las limitaciones que los adultos mayores tienen, de acuerdo a una guía específica para este tipo de usuarios (Silva et al., 2015).

Consta de una interfaz muy simple, que se divide en 3 partes:

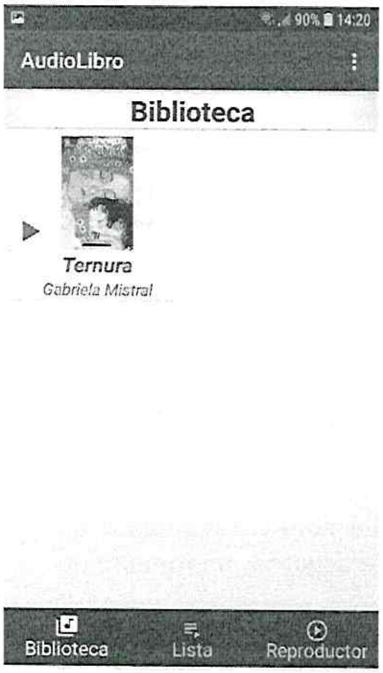

Figura 1: Biblioteca

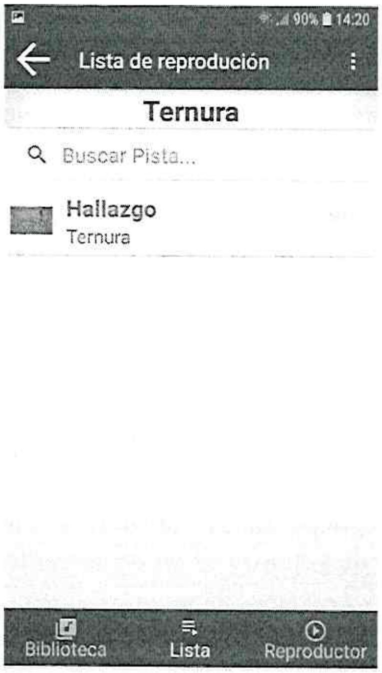

Figura 2: Lista Reproducción

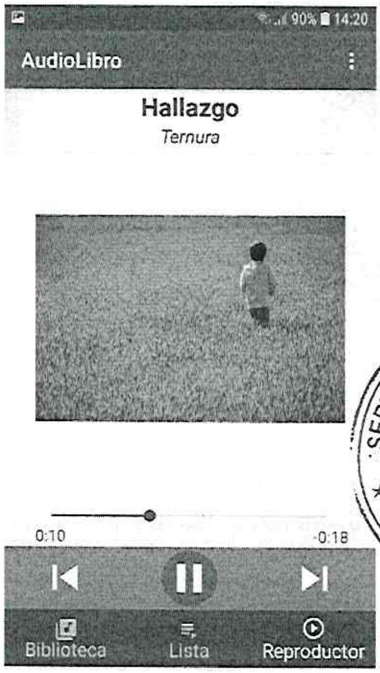

Figura 3: Reproductor

En la Figura 1 se podrá seleccionar el libro que se desea reproducir. En la Figura 2, considerando el libro seleccionado, se podrá elegir el poema (para este caso) que se quiere escuchar de todos los disponibles. Por último, en la Figura 3 se podrá detener o volver a reproducir el poema seleccionado.

Esta aplicación recabará de forma automática y sin la intervención del adulto mayor, información de su uso, y dicha información será almacenada de forma segura en un servidor externo a la aplicación, respetando la confidencialidad de los datos del usuario utilizando un código para representar cada uno de los participantes. Dicha información será la siguiente:

- identificación del adulto mayor,
- fecha, hora y duración de cada reproducción,
- pistas (poemas) escuchadas.

Respecto de esta información se medirá como variable independiente la usabilidad (cuan amigable es la aplicación para el usuario) y la frecuencia de uso (cuanto uso se hace diariamente de la aplicación).

A continuación, se detallan las variables a nivel operacional

- A continuación, se realizará una definición operacional de las variables de la presente investigación con la finalidad de clarificar los conceptos a abordar.

1. **Las características sociodemográficas**:
   a. Edad: Variable independiente, escala de razón. En análisis posterior es posible que se categorice en rangos.
   b. Sexo: Variable independiente nominal dicotómica: mujer / hombre.
   c. Escolaridad: Variable independiente, escala de razón. Años de estudios completados de educación formal. En análisis posterior es posible que se categorice en: analfabeto, educación básica incompleta, educación básica

SERVICIO DE SALUD CONCEPCIÓN
COMITÉ ÉTICO CIENTÍFICO
04 MAYO 2021
CONCEPCIÓN

completa, educación media incompleta, educación media completa, educación superior completa e incompleta.

d. Competencias digitales: Variable independiente, ordinal (ausencia, presencia). La competencia digital es el conjunto de conocimientos, habilidades, actitudes que se requieren cuando se utilizan las TICs y los medios digitales para realizar tanto tareas, resolver problemas, comunicar y gestionar la información como construir conocimiento de manera eficiente, apropiada y autónoma. Esta competencia se puede medir a través de un cuestionario (anexo 4). Consta de 20 ítems donde se debe indicar si se puede o no desarrollar la actividad indicada.

2. **Bienestar eudaimónico:** Variable dependiente ordinal, entendida como totalmente en desacuerdo, en desacuerdo, ni de acuerdo ni en desacuerdo, de acuerdo, totalmente de acuerdo.
3. **Bienestar hedónico:** Variable dependiente ordinal, entendida como totalmente en desacuerdo, en desacuerdo, ni de acuerdo ni en desacuerdo, de acuerdo, totalmente de acuerdo.
4. **Bienestar social:** Variable dependiente ordinal, entendida como totalmente en desacuerdo, en desacuerdo, ni de acuerdo ni en desacuerdo, de acuerdo, totalmente de acuerdo.

Todas las variables de bienestar serán procesadas en análisis posterior como una escala única de bienestar (escala de razón), post exploración de su validez.

En la parte inicial el rango de puntaje va de 0 a 30 puntos Parte A.

En la aplicación final el puntaje va de 0 a 60 puntos Parte A + B.

El rango de puntajes es distinto porque se agrega la parte B que mide cambios específicos en la aceptación de la tecnología en la cotidianeidad post uso de la aplicación.

5. **Aplicación móvil audiolibro:** Variable independiente.
   a. **Usabilidad:** Variable independiente, puntaje escala de razón (rango de 0 a 100 puntos). La usabilidad es términos simples la facilidad con que los usuarios pueden utilizar un software. Esta facilidad se puede medir con el cuestionario *System Usability Scale* o SUS (anexo 3 para ver las preguntas (escala Likert)). Este test consta de 10 ítems con cinco opciones de respuesta para los encuestados, las que varían desde “totalmente en desacuerdo” a “totalmente de acuerdo”.
   b. **Frecuencia de uso:** Variable independiente, escala de razón. Cantidad de audios escuchados al día.

3.4 Población y muestra

Población de donde provendrán los participantes:

La población corresponde a adultos mayores (AM) a partir de los 60 años que se atienden en atención primaria en el establecimiento del Centro Comunitario de Rehabilitación, de la comuna de Concepción. La comuna Concepción tiene 223.574 habitantes de los cuales el 12,46% correspondiente a 27.858 son AM (Instituto Nacional de Estadísticas, 2017).

El Centro Comunitario de Rehabilitación de Concepción (CCR) atiende a personas con patologías GES de origen neurológico y no neurológico. Las atenciones se encuentran basadas en la Rehabilitación con base comunitaria, por lo que se realizan actividades de forma individual y grupal, además de educación en promoción en salud y prevención de

enfermedad para la comunidad, visitas domiciliarias, intervención específica por profesionales Kinesiólogo y Terapeutra Ocupacional del centro, centro formador en convenio asistencial docente con la Carrera de Fonoaudiología, Terapia Ocupacional, Kinesiología y trabajo en red. Actualmente atiende a usuarios que se encuentran inscritos en los CESFAM O'Higgins, Juan Soto Fernández y Tucapel de la comuna. Se informa que el año 2019 ingresaron 250 adultos mayores a CCR y en el año 2018 fueron 320; los datos señalados fueron facilitados por la Gestora del CCR Concepción, la kinesióloga Nadia Muñoz.

Criterios de selección de participantes: Inclusión-Exclusión

Criterios de Inclusión:

- Persona de 60 años en adelante que pertenezca al CCR Concepción.
- Que se ofrezca voluntariamente a participar en el estudio confirmado a través de la firma del consentimiento informado.
- Hablante de español.
- Habilitado y capaz de aprobar el consentimiento informado.
- Que posea un smartphone donde ejecutar la aplicación móvil de audiolibro.
- Adultos mayores que no se encuentren participando en otro programa, tanto del aspecto emocional, social y/o motor.

SERVICIO DE SALUD CONCEPCION
COMITÉ ÉTICO CIENTÍFICO
04 MAYO 2021
CONCEPCIÓN

Criterios de Exclusión

- Enfermedad psiquiátrica o médica significativa (trastorno depresivo, delirium, discapacidad intelectual, etc).
- Presencia de enfermedades neurodegenerativas (demencia, enfermedad de Alzheimer, enfermedad de Parkinson, esclerosis múltiple, etc).
- Adulto Mayor catalogado con criterio de dependencia.
- Presencia de un déficit sensorial severo, ya sea en dificultades visuales o auditivas.
- Personas analfabetas.

Cálculo y tamaño de la muestra

El tipo de muestreo es no probabilístico por conveniencia, el modo de reclutamiento de los participantes se explica a continuación. Brevemente, se invitará a las personas que se atiendan en CCR que cumplan con los criterios de inclusión y exclusión, y que deseen participar a través de convocatoria abierta. Se ha estimado un máximo de 60 participantes considerando el estudio de Ameri et al., (2017), en el cual la muestra fue de 60 personas que accedieron de forma voluntaria a participar. Por otro parte en un estudio de Poerio y Totterdell (2020), el tamaño de la muestra fue de 90 participantes que fueron invitados a participar de manera voluntaria. Frente a estos antecedentes parece apropiado el número que se ha fijado como máximo. Respecto al mínimo necesario para hacer la evaluación pre y post uso de la app, se ha considerado un mínimo de 30 participantes que permita obtener datos de los distintos quintiles y género dentro de la muestra.

Descripción de proceso de reclutamiento de los participantes

Posterior a la autorización del jefe de la DASM de Concepción y jefe administrativo del CCR Concepción se ejecutará el reclutamiento de la siguiente forma:

Se ubicarán afiches en el mural del CCR para realizar una convocatoria abierta a todas las personas mayores de 60 años que se atiendan en este lugar y que deseen participar de la investigación. El afiche tendrá escrito el lugar al que deben asistir dentro de las dependencias del centro para inscribirse. Cuando soliciten la inscripción se les pedirá nombre completo y teléfono para poder contactarlo.

Además, se ejecutarán reuniones grupales programadas por los encargados del proyecto en dependencias del CCR, para realizar la invitación a participar en la investigación.

Una vez que los interesados se hayan inscrito, se comenzará a contactar vía telefónica a los sujetos para invitarlos a participar de manera voluntaria, en esta instancia se entregará la información de forma clara y concisa, indicado los objetivos de la investigación y la estrategia que se utilizará. En esta instancia se realizarán preguntas claves para determinar si presenta los criterios de inclusión al estudio y se confirmará la participación.

Si el usuario rehúsa a participar, se agradecerá y se indica que no existe perjuicio alguno.

En caso de aceptar, se citará al CCR en un horario a convenir y se le explicará el consentimiento informado de manera verbal con apoyo del documento escrito (ver consentimiento informado en Anexo 1), recalcando que se trata de un estudio de carácter voluntario en el cual se resguardará y asegurará la confidencialidad de su información personal.

Si asiente se realizará la firma de dicho documento (entregando una copia al participante) y se continuará de inmediato con la aplicación de las encuestas y cuestionarios previos al uso de la aplicación.

En la oficina del CCR, uno de los encargados del proyecto se hará cargo de explicar la modalidad de respuesta de los cuestionarios, lo que son autoaplicables. Se acompañará al usuario mientras contesta los cuestionarios por si surge alguna duda. Se aplicarán cuestionario de destrezas digitales y preguntas previa a la experiencia del cuestionario sobre el bienestar en experiencia audiolibro para personas mayores.

Posteriormente se explicará el funcionamiento de la aplicación de audiolibro, se entregará un número de contacto para resolver las consultas que puedan surgir. Dos semanas después de la entrega de la aplicación, se les contactará para monitorear el proceso. Al mes de uso se les citará para realizar la aplicación del cuestionario de bienestar y el cuestionario de usabilidad.

SERVICIO DE SALUD CONCEPCIÓN
COMITÉ ÉTICO CIENTÍFICO
0 4 MAYO 2021

Luego de realizada la evaluación se llevará a cabo el análisis de los datos.

3.5 Instrumento de recogida de información

Es importante señalar que se aplicará la medición en un box del dispositivo de salud, con buena iluminación, disponibilidad de sillas y sin distractores, en el horario acordado previamente en la llamada telefónica de contacto. Serán los investigadores quienes realizarán las evaluaciones previa y posterior al uso de aplicación.

A continuación, se presentan los instrumentos de recogida de información.

Cuestionario sobre el Bienestar en la experiencia audiolibro para las personas mayores

El bienestar será valorado a través de un cuestionario sobre el bienestar en la experiencia de audiolibro para personas mayores, creado por los investigadores para este estudio. Busca conocer sobre el bienestar en los adultos mayores previo y posterior al uso de la aplicación móvil. Presenta una etapa A que contiene 6 preguntas, que se aplica previo al uso de la aplicación y una parte B, que está compuesta de 12 preguntas y se aplica posterior a

uso de la aplicación móvil (Anexo 2). Las preguntas se encuentran todas en escala Likert de Totalmente en desacuerdo (0) Totalmente de acuerdo (5), especificando un puntaje mínimo para la Parte A de 0 puntos a un máximo de a 30 puntos, y para la parte B de un mínimo de 0 puntos a un máximo de 60 puntos.

Para la adaptación del lenguaje del cuestionario, se va a seleccionar un grupo pequeño de 5 a 10 adultos mayores para que revisen el modo en que está escrito el instrumento y lo retroalimenten. La idea es que si hay conceptos o una redacción que no entiende o si las palabras utilizadas no están adaptadas a la realidad local y grupal, estas personas puedan sugerirnos cambios que serán adoptados.

Cuestionario para medir la Usabilidad de una Aplicación (SUS)

El *System Usability Scale* (SUS) fue inventado por John Brooke quien, en 1986, creó esta escala de usabilidad "rápida y simple" para evaluar prácticamente cualquier tipo de sistema o aplicación (Brooke, 1996).

El cuestionario SUS ocupa una escala de likert y está compuesto por 10 preguntas cuidadosamente seleccionadas, en las cuales el encuestado selecciona el grado de "acuerdo" o "desacuerdo" con el ejemplo propuesto y evalúa ésta en una escala de cinco puntos, donde 1 significa "Totalmente en desacuerdo" y 5 significa "Totalmente de acuerdo".

Para calcular la puntuación del SUS, hay que sumar primero las contribuciones de cada ítem. La contribución de cada ítem valdrá entre 0 y 4.

Para los ítems 1, 3, 5, 7 y 9, la contribución será la posición de la escala menor a 1. Para los ítems 2, 4, 6, 8 y 10, la contribución será 5 menos la posición en la escala. Se multiplica la suma de los resultados por 2,5 para obtener el valor global del SUS. El resultado estará entre 0 y 100, donde mayor sea el resultado, mayor es el nivel usabilidad del sistema o aplicación.

En el Anexo 3 se presenta el cuestionario para medir la usabilidad de una aplicación.

Cuestionario para medir Competencias Digitales

La competencia digital ha sido reconocida como una de las 8 competencias clave para el aprendizaje permanente de la Unión Europea. La competencia digital se puede definir ampliamente como el uso seguro, crítico y creativo de las TICs para alcanzar los objetivos relacionados con el trabajo, la capacidad de emprender, el aprendizaje, el ocio, la inclusión y/o la participación en la sociedad (Ferrari, 2012).

En el cuestionario para medir competencias digitales (DIGCOMP por su traducción al inglés) (Ferrari, 2012), se identifican cuatro áreas de competencias relevantes: información, comunicación, creación de contenidos y resolución de problemas. El cuestionario se evalúa considerando 3 niveles: **Ninguno**, **Básico**, y **Sobre básico**, donde:

- **Ninguno**: Si la persona no puede realizar ninguno de los ítems del área.
- **Básico**: Si la persona logra realizar solo uno de los ítems del área.
- **Sobre básico**: Si la persona puede realizar al menos dos de los ítems del área.

SERVICIO DE SALUD CONCEPCIÓN
COMITÉ ÉTICO CIENTÍFICO
0 4 MAYO 2021

A cada una de las áreas se le asigna una de estas etiquetas según las respuestas. Luego para evaluar el cuestionario y obtener un indicador global de los resultados, éstos se evalúan de la siguiente manera:

- **No**: Se refiere a las personas que no respondieron ninguna en todas las áreas, además de aquellos que no han utilizado Internet en los últimos 12 meses o que nunca han utilizado Internet.
- **Bajo**: Se refiere a las personas que tienen uno o más "ninguno" en tres áreas.

- **Básico**: Se refiere a las personas que tienen uno o más "básico", pero no "ninguna".
- **Sobre básico**: Se refiere a las personas con "sobre básico" en las cuatro áreas.

En el Anexo 4 se presenta el cuestionario para medir competencias digitales.

Proceso de recolección de información y su manejo

Proceso de recogida de datos: Se realizará una reunión inicial en la que se le aplicarán los cuestionarios para medir el bienestar parte A del cuestionario y las destrezas digitales del participante. Se hará una inducción para el uso de la aplicación, se instalará la aplicación de audiolibro en el celular y se le volverá a citar en un plazo de un mes para aplicar la encuesta de bienestar parte B y el cuestionario de usabilidad. Para la aplicación de los instrumentos a cada participante se le asignará un código para proteger y resguardar sus datos personales.

Manejo de datos: La base de datos será confidencial y de manejo único por los investigadores, cada participante se identificará con un código administrativo para salvaguardar la confidencialidad de la información.

3.6 Propuesta de análisis de datos

Para el análisis de los resultados se confeccionará una base de datos única con los resultados tabulados de los instrumentos y los datos de caracterización sociodemográfica de los participantes. Posteriormente se utilizará estadística descriptiva mediante el uso de análisis de frecuencias uni y bivariados y medidas de tendencia central (media y desviación estándar) para la descripción de las características de la muestra y los resultados de los cuestionarios.

En una segunda etapa se someterán a prueba de hipótesis del estudio mediante una regresión lineal multivariada para predecir la variable bienestar al final de la intervención, explicada por bienestar inicial, y controlada por variables sociodemográficas, usabilidad y frecuencia de uso. Se explorara si las covariables cumplen un rol de confundentes y/o efectos modificadores en la relación entre el puntaje inicial y el puntaje final en bienestar. El modelo en específico se presupuesta sea Modelo Lineal Generalizado, para distribución normal y link identidad. El procesamiento y análisis de los datos se realizara con software IBM Statistical Package for Social Sciences (SPSS).

El modelo estadístico presupuestado se puede resumir como:

$$Bfinal = a_0 + b\ (Binicial) + b(frec) + b\ (usabilidad) + b(sexo) + b\ (otras\ dem)$$

Dónde:
Bfinal: puntaje de bienestar final,
a_0 ; intercepto
Binicial: pendiente bienestar inicial
b(frec): pendiente asociada a la frecuencia de uso de la app
b(usabilidad): pendiente asociada a la usabilidad evaluada de la app
b(sexo): sexo del participante
b(otras dem): pendientes asociadas a otras variables demográficas que resulten confundentes o efectos modificadores relevantes.

SERVICIO DE SALUD CONCEPCIÓN
COMITÉ ÉTICO CIENTÍFICO
0 4 MAYO 2021
CONCEPCIÓN

3.7 Consideraciones éticas

El presente estudio corresponde a un proyecto de investigación realizado en conjunto entre el Departamento de Fonoaudiología de la Universidad de Concepción y el Departamento

de Ingeniería Informática de la Universidad Católica de la Santísima Concepción y cumplirá con las Buenas Prácticas Clínicas dadas por el Gobierno de Chile. Se resguardará el cumplimiento de la Ley de derechos y deberes del paciente (Ley 20.584) y se incluirán todos los aspectos de recopilación y manejo de la información. Además, la investigación a realizar se encuentra bajo las normas éticas de la declaración de Helsinki.

La información que se solicitará a los participantes corresponde a: consentimiento informado, aplicación de cuestionarios de bienestar, cuestionario para medir la usabilidad de una aplicación (SUS), y cuestionario para medir competencias digitales (DIGCOMP). El cuestionario de bienestar se realizará antes (parte A) y después del uso (parte B) de la aplicación móvil, el DIGCOMP previo al uso y el SUS posterior a la experiencia. Los datos recopilados y entregados por los participantes son extremadamente valiosos y confidenciales, por tanto, solo miembros del equipo investigador tendrán acceso a la información. Se resguardará la privacidad y para esto, a cada participante se le asignará un código administrativo, de manera que la identidad se resguarde.

Para cautelar la ética en la ejecución de la investigación se contemplaron los "Requisitos éticos de investigación en seres humanos" de acuerdo a lo siguiente (Rodríguez Yunta, 2004)):

- Participarán en la investigación los usuarios que asientan voluntariamente su participación en la investigación, y para evidenciar dicho proceso se solicitará la firma de un consentimiento informado.
- Consentimiento informado: cada uno de los participantes invitados decidirán de manera voluntaria su participación, el consentimiento será en modalidad verbal y escrita, en el cual se detallará el procedimiento y las implicancias de la participación en la investigación, orientado en los principios éticos de la declaración de Helsinki, se responderá a cualquier pregunta que presente el participante y se le entregará una copia del consentimiento informado con un número de contacto frente a cualquier inquietud que pueda surgir. La participación es de carácter voluntario y se resguardará la información, es decir, se tomarán toda clase de precauciones para resguardar y respetar la intimidad de la persona participante y la confidencialidad de su información personal, esto a través del uso de códigos para referirse al sujeto participante. La investigación no tiene ningún costo asociado para los participantes. Los beneficios que se esperan encontrar corresponden a un cambio o mantención de su bienestar, además de ser una herramienta que los acerque a la cultura a través del uso de herramientas tecnológicas. En cuanto al ámbito social, se espera que sea una herramienta que colabore a la salud integral de los adultos mayores. El uso de los resultados de la investigación serán relevantes para evidenciar los beneficios de la aplicación en la salud integral del adulto mayor, y poder promover este el uso de este tipo de herramientas. En conclusión, el proceso del consentimiento informado estará fundado en los siguientes pilares: entregar información clara, comprensible y detallada, asegurar la comprensión por parte del participante, el libre albedrío del sujeto y el espacio para responder a todo tipo de dudas que se necesite resolver.
- Respeto a los sujetos inscritos: Los investigadores garantizarán la confidencialidad de los participantes y además respetarán el derecho del usuario a retirarse del estudio cuando el amerite sin perjuicio alguno.

SERVICIO DE SALUD CONCEPCIÓN
COMITÉ ÉTICO CIENTÍFICO
04 MAYO 202[illegible]
CONCEPCIÓN

Con respecto a las implicancias éticas del proyecto, el equipo investigador manifiesta estar consciente de que el uso de la información, la privacidad y confidencialidad del sujeto y sus datos, pues es información sensible, y su uso será solo para los objetivos descritos y acotado a lo expresado en el presente documento. Es el participante quien

entregará permiso a través de su firma en el consentimiento informado para el acceso de la información, basado en la ley 20.120 y 20.584.

4. Plan de trabajo

Debido a la contingencia mundial, no es posible detallar acciones específicas.

Actividades	Plazos
Generar y validar lenguaje de la encuesta para aplicar a adultos mayores.	Mes 1
Desarrollo de la aplicación.	Mes 3 al mes 4
Crear un Protocolo de investigación para "Audiolibro".	Mes 2
Presentar proyecto de investigación al CEC del Servicio de Salud Concepción.	Mes 2 y mes 3
Proporcionar el contenido que se utilizará en la aplicación de "Audiolibro" para adultos mayores.	Mes 3 y Mes 4
Realizar la prueba piloto de la aplicación.	Mes 4 al mes 5
Realizar convocatoria a los participantes.	Mes 6
Aplicar Cuestionarios.	Mes 7
Monitorear el uso de la aplicación "Audiolibro" en la muestra.	Mes 8
Aplicar las encuestas.	Mes 9
Recopilar datos del uso de "Audiolibro".	Mes 10
Analizar los datos obtenidos.	Mes 11
Redactar un escrito para publicación.	Mes 11 al 15
Difundir de manera gratuita la aplicación.	Mes 13 y 14
Mantener la plataforma Audiolibro y actualizaciones posteriores.	Mes 14 al mes 24

SERVICIO DE SALUD CONCEPCIÓN
COMITÉ ÉTICO CIENTÍFICO
0 4 MAYO 2021
CONCEPCIÓN

5. Bibliografía

Abello, R., Amarís, M., Blanco, A., Madariaga, C., Díaz, D., & Arciniégas, T. (2008). Bienestar, autoestima, depresión y anomia en personas que no han sido víctimas de

violencia política y social. *Investigación & Desarrollo*, *16*(2).

Albala, C. (2020). EL ENVEJECIMIENTO DE LA POBLACIÓN CHILENA Y LOS DESAFÍOS PARA LA SALUD Y EL BIENESTAR DE LAS PERSONAS MAYORES. *Revista Médica Clínica Las Condes*, *31*(1). https://doi.org/10.1016/j.rmclc.2019.12.001

Alstergren, I., Andersson, A., Hedman, L., & Maric, D. (2020). *DreamScape, a Platform for Creating, Sharing and Listening to Interactive Stories*. Uppsala University.

Ameri, F., Vazifeshenas, N., & Haghparast, A. (2017). The impact of audio book on the elderly mental health. *Basic and Clinical Neuroscience*, *8*(5). https://doi.org/10.18869/NIRP.BCN.8.5.361

Blanco, A., & Díaz, D. (2005). Social well-being: Theoretical structure and measurement. *Psicothema*, *17*(4).

Brooke, J. (1996). SUS: A quick and dirty usability scale. In *Usability Evaluation In Industry* (pp. 189–194).

Casamayou, A., & Morales González, M. J. (2018). Elderly people and digital technologies: a dual challenge Idosos e tecnologias digitais: desafios duplos. *Conocimiento y Sociedad*, *7*(2), 199–226.

Diener, E., & Suh, E. M. (2000). This excerpt from Culture and Subjective Well-Being. In *books.google.com*.

Endrstova, B., Macik, M., & Treml, L. (2018). Reprobooktor: A concept of audiobook player for visually impaired older adults. *Proceedings of the 9th IEEE International Conference on Cognitive Infocommunications (CogInfoCom 2018)*, 63–68.

Fang, Y., Chau, A. K. C., Wong, A., Fung, H. H., & Woo, J. (2018). Information and communicative technology use enhances psychological well-being of older adults: the roles of age, social connectedness, and frailty status. *Aging and Mental Health*, *22*(11). https://doi.org/10.1080/13607863.2017.1358354

Ferrari, A. (2012). *Digital competence in practice: an analysis of frameworks*. Reporte Técnico, Research Centre of the European Commission.

Fletcher, J., & Jensen, R. (2016). Overcoming barriers to mobile health technology use in the aging population. *Online Journal of Nursing Informatics*, *19*(3), 1.

Gallardo-Peralta, L. P., & Sánchez-Moreno, E. (2019). Successful aging and personal well-being among the chilean indigenous and non-indigenous elderly*. *Aquichan*, *19*(3). https://doi.org/10.5294/aqui.2019.19.3.9

García-herrera, D. G., Cárdenas-cordero, N. M., & Erazo-álvarez, J. C. (2020). *Comprensión lectora e innovación educativa: estrategias para mejorar la lectoescritura en los jóvenes del bachillerato*. *VI*, 337–363. https://doi.org/10.35381/cm.v6i1.337

García-Rodríguez, A., & Gómez-Díaz, R. (2019). ¿Leer con los oidos?: audiolibros y literatura infantil y juvenil. *Anuario ThinkEPI*, *13*. https://doi.org/10.3145/thinkepi.2019.e13c01

García, C., & González, I. (2000). LA CATEGORÍA BIENESTAR PSICOLÓGICO Y SU RELACIÓN CON OTRAS CATEGORÍAS SOCIALES. *Revista Cubana de Medicina General Integral*, *16*(6).

Guaña-Moya, E. J., Quinatoa-Arequipa, E., & Pérez-Fabara, M. A. (2017). Tendencias del uso de las tecnologías y conducta del consumidor tecnológico. *Ciencias Holguín*, *23*(2), 15–30.

Hernández, R., Fernández, C., & Baptista, P. (2014). Metodología de la investigación. In *Journal of Chemical Information and Modeling* (Vol. 53, Issue 9). https://doi.org/10.1017/CBO9781107415324.004

Instituto Nacional de Estadísticas. (2017). *Compendio Estadístico (Statistical Compendium)*. www.ine.cl

ISO 9241-11. (2018). *Ergonomics of human-system interaction — Part 11: Usability:*

SERVICIO DE SALUD CONCEPCIÓN
COMITÉ ÉTICO CIENTÍFICO
0 4 MAYO 2021
CONCEPCIÓN

Definitions and concepts. https://www.iso.org/obp/ui/#iso:std:iso:9241:-11:ed-2:v1:en

Keyes, C. L. M., Shmotkin, D., & Ryff, C. D. (2002). Optimizing well-being: The empirical encounter of two traditions. *Journal of Personality and Social Psychology*, *82*(6). https://doi.org/10.1037/0022-3514.82.6.1007

Khosravi, P., Rezvani, A., & Wiewiora, A. (2016). The impact of technology on older adults' social isolation. *Computers in Human Behavior*, *63*, 594–603. https://doi.org/10.1016/j.chb.2016.05.092

Kim, J., Lee, H. Y., Candace Christensen, M., & Merighi, J. R. (2017). Technology access and use, and their associations with social engagement among older adults: Do women and men differ? In *Journals of Gerontology - Series B Psychological Sciences and Social Sciences* (Vol. 72, Issue 5). https://doi.org/10.1093/geronb/gbw123

Kreps, G. L., & Neuhauser, L. (2010). New directions in eHealth communication: Opportunities and challenges. *Patient Education and Counseling*, *78*(3), 329–336.

Larson, L. C. (2015). E-Books and Audiobooks: Extending the Digital Reading Experience. *The Reading Teacher*, *69*(2), 169–177. https://doi.org/10.1002/trtr.1371

Leiton Espinoza, Z. E. (2016). El envejecimiento saludable y el bienestar: un desafío y una oportunidad para enfermería. *Enfermería Universitaria*, *13*(3). https://doi.org/10.1016/j.reu.2016.06.002

Loh, K. Y., & Ogle, J. (2004). Age related visual impairment in the elderly. In *Medical Journal of Malaysia* (Vol. 59, Issue 4).

Macik, M., Maly, I., Balata, J., & Mikovec, Z. (2017). How can ICT help the visually impaired older adults in residential care institutions: The everyday needs survey. *Proceedings of the 8th IEEE International Conference on Cognitive Infocommunications (CogInfoCom 2017)*, 157–164.

Martínez Fuentes, A. J., & Fernández Díaz, I. E. (2008). Ancianos y salud. *Revista Cubana de Medicina General Integral*, *24*(4).

Matas-Terrón, A., Leiva Olivencia, J. J., & Franco Caballero, P. D. (2016a). PREVISIÓN DE NECESIDADES FORMATIVAS PARA UN ENVEJECIMIENTO ACTIVO. *Píxel-Bit, Revista de Medios y Educación*, *48*. https://doi.org/10.12795/pixelbit.2016.i48.15

Matas-Terrón, A., Leiva Olivencia, J. J., & Franco Caballero, P. D. (2016b). PREVISIÓN DE NECESIDADES FORMATIVAS PARA UN ENVEJECIMIENTO ACTIVO. *Píxel-Bit, Revista de Medios y Educación*, *48*, 225–240. https://doi.org/10.12795/pixelbit.2016.i48.15

Mora Quezada, J. de las N., Osses Paredes, C. F., & Rivas Arenas, S. M. (2017). Funcionalidad del adulto mayor de un Centro de Salud Familiar Hualpén-Chile. *Rev. Cuba. Enferm*, *33*(1), 18–30. http://scielo.sld.cu/scielo.php?script=sci_arttext&amp%0Apid=S0864-03192017000100004%0Ahttp://www.revenfermeria.sld.cu/index.php/enf/article/view/432

Moyer, J. E. (2012). Audiobooks and E-books: A Literature Review. *Reference & User Services Quarterly*, *51*(4), 340–354.

Organización Mundial de la Salud (OMS). (2006). *Constitución de la Organización Mundial de la Salud. 45. Ginebra. Recuperado el Noviembre de 2015.* 1. http://www.who.int/governance/eb/who_constitution_sp.pdf.

Ortiz Arriagada, J. B., & Castro Salas, M. (2009). The psychological well-being of the elderly and their relation with self-esteem and self-efficiency: Nursing contribution. *Cienc. Enferm*, *15*(1). https://doi.org/10.4067/S0717-95532009000100004

Poerio, G., & Totterdell, P. (2020). The Effect of Fiction on the Well-Being of Older Adults: A Longitudinal RCT Intervention Study Using Audiobooks. *Psychosocial Intervention*, *29*(1), 29–37.

SERVICIO DE SALUD CONCEPCIÓN
COMITÉ ÉTICO CIENTÍFICO
0 4 MAYO 2024

Rodríguez,Y.Negrón, N. Maldonado, Y., Quiñones, & A. Osorio, N. (2015). *Dimensiones de bienestar psicológico y apoyo social percibido con relación al sexo y nivel de estudio universitario*. *33*(1), 31–43. http://www.scielo.org.co/pdf/apl/v33n1/v33n1a03.pdf

Rodríguez Yunta, E. (2004). Comités De Evaluación Ética Y Científica Para La Investigación En Seres Humanos Y Las Pautas Cioms 2002. *Acta Bioethica*, *10*(1), 37–47. https://doi.org/10.4067/S1726-569X2004000100005

Romero Carrasco, A., Brustad, R., & Garcia Mas, A. (2007). Bienestar psicológico y su uso en la psicología del ejercicio, la actividad física y el deporte. *Revista Iberoamericana de Psicología Del Ejercicio y El Deporte*, *2*(2).

Ronquillo, C. C., & Peña, J. M. (2017). El audiolibro como herramienta tecnológica para el aprendizaje de los estudiantes de la carrera de bibliotecología y archivología de la Universidad de Guayaquil. *Espirales: Revista Multidisciplinaria de Investigación*, *1*(10). https://doi.org/10.31876/re.v1i10.207

Rubin, J., & Chisnell, D. (2008). *Handbook of Usability Testing* (2da ed.). Wiley Publishing, Inc.

Silva, P. A., Holden, K., & Jordan, P. (2015). Towards a List of Heuristics to Evaluate Smartphone Apps Targeted at Older Adults: A Study with Apps that Aim at Promoting Health and Well-Being. *Proceedings of the 48th Hawaii International Conference on System Sciences*, 3237–3246.

Smallfield, S., & Kaldenberg, J. (2020). Occupational Therapy Practice Guidelines for Older Adults With Low Vision. *The American Journal of Occupational Therapy*, *74*(2), 7402390010.

Villafuerte, J., Yenny, R., Abatt, A., Alonso, Y., Yuleydi, V., Guardado, A., & Leyva, I. (2017). Elderly well-being and quality of life , a challenge for inter-sectoral action. *Medisur*, *1*.

Vukovic, V., Favaretti, C., Ricciardi, W., & De Waure, C. (2018). HEALTH TECHNOLOGY ASSESSMENT EVIDENCE on E-HEALTH/M-HEALTH TECHNOLOGIES: EVALUATING the TRANSPARENCY and THOROUGHNESS. *International Journal of Technology Assessment in Health Care*, *34*(1). https://doi.org/10.1017/S0266462317004512

Zhang, D., & Adipat, B. (2005). Challenges, Methodologies, and Issues in the Usability Testing of Mobile Applications. *International Journal of Human-Computer Interaction*, *18*(3), 293–308.

Zubieta, E., & Delfino, G. (2010). Satisfacción con la vida, bienestar psicológico y bienestar social en estudiantes universitarios de Buenos Aires. *Anuario de Investigaciones*, *17*(1).

SERVICIO DE SALUD CONCEPCIÓN
COMITÉ ÉTICO CIENTÍFICO
04 MAYO 2021
CONCEPCIÓN

Anexo 1

CONSENTIMIENTO INFORMADO PARA INVESTIGACIÓN CLÍNICA

Título de la investigación: Uso de aplicación de audiolibro en adultos mayores pertenecientes al Centro Comunitario de Rehabilitación y el impacto en su bienestar.

Usted ha sido invitado a participar en la investigación "Uso de aplicación de audiolibro en adultos mayores pertenecientes al Centro Comunitario de Rehabilitación y el impacto en su bienestar".

El objetivo de esta investigación es determinar los cambios en la percepción del bienestar de los adultos mayores que se atienden en CCR Concepción, posterior al uso de una aplicación móvil para escuchar audios de poemas.

Para decidir participar en esta investigación es importante considerar la siguiente información:

Participación: su participación consistirá en responder de forma personal 2 cuestionarios, utilizar la aplicación de audiolibro para la escucha de poemas por 1 mes y al terminar ese periodo, responder 2 cuestionarios más. En una fecha a su convenir será citado al CCR Concepción por los investigadores responsables para realizar estos cuestionarios. La duración de la investigación será de 6 semanas aproximadamente.

Beneficios: no recibirá ningún beneficio directo, ni recompensa alguna por participar en este estudio, ni compensación económica. No obstante, su participación permitirá generar información para determinar si hay cambios en

SERVICIO DE SALUD CONCEPCIÓN
COMITÉ ÉTICO CIENTÍFICO
04 MAYO 2021
CONCEPCIÓN

la percepción del bienestar en el adulto mayor posterior a la escucha de poemas en la aplicación de audiolibro.

Riesgos: no genera ningún tipo de daño, no obstante, la manipulación de la aplicación en su teléfono podría aumentar el consumo de datos móviles. El tiempo de aplicación de las encuestas, de aproximadamente 1 hora, podría generarle fatiga.

Voluntariedad: su participación es absolutamente voluntaria. Tiene la libertad de contestar las preguntas que desee, decidir no participar, como también de detener su participación en cualquier momento. Esto no implicará ningún perjuicio para usted.

SERVICIO DE SALUD CONCEPCIÓN
COMITÉ ÉTICO CIENTÍFICO
0 4 MAYO [illegible]

Confidencialidad: todas sus opiniones, respuestas y datos serán confidenciales y mantenidas en estricta reserva. Su nombre no aparecerá asociada a la investigación en ningún momento. Toda la información y datos serán custodiados únicamente por los investigadores responsables a través de un código numérico para la identificación de cada uno de los participantes, de esta forma no se utilizan los datos personales.

Conocimiento de los resultados: usted tiene derecho a conocer los resultados de la investigación. Si desea conocer los resultados globales de la investigación puede solicitarlos a los miembros del equipo de investigación. En el caso de obtener algún resultado de relevancia clínica, el equipo de investigadores se compromete a informar al participante y al equipo de salud tratante.

Datos de contacto: sienta la libertad de realizar las preguntas que desee en cualquier momento de su participación al correo electrónico de los investigadores responsables.

Flga. Valeria Espejo Videla; mail: valeriaespejo@udec.cl
Flga. Laura Aravena Canese; mail: laravena@udec.cl
Dr. Pedro Rossel Cid; mail: prossel@ucsc.cl
Contacto: +56983375946
Dirección: Janequeo sin número esquina Chacabuco. Facultad de Medicina, 3er piso, Departamento de Fonoaudiología.

*Frente a cualquier duda o comentario acerca del presente estudio contactar a Comité Ético Científico del Servicio de Salud Concepción presidido por Dr. Nelson Pérez Terán. **Email:** cec@ssconcepcion.cl. **Fono**: 56-41-2722745, red. Minsal 412745. **Dirección**: Hospital Guillermo Grant Benavente. San Martin 1436, Concepción. **Página Web**: http://cec.dssc.cl

SERVICIO DE SALUD CONCEPCION
COMITE ETICO CIENTIFICO
0 4 MAYO 2021
CONCEPCION

FORMULARIO DE CONSENTIMIENTO INFORMADO

Título de la investigación: Uso de aplicación de audiolibro en adultos mayores pertenecientes al Centro Comunitario de Rehabilitación y el impacto en su bienestar.

Yo,____, acepto participar voluntariamente en el estudio denominado "Uso de aplicación de audiolibro en adultos mayores pertenecientes al Centro Comunitario de Rehabilitación y el impacto en su bienestar".

Declaro que he leído y comprendido en qué consiste mi participación en este estudio y que puedo retirarme si así lo deseo.

____

Participante

Nombre participante: ____________________________ Fecha: Firma:

Nombre de persona que realiza el consentimiento: _______________

Fecha: Firma:

SERVICIO DE SALUD CONCEPCIÓN
COMITÉ ÉTICO CIENTÍFICO
04 MAYO 2021
CONCEPCIÓN

Concepción, abril 2021.

Anexo 2

CUESTIONARIO SOBRE EL BIENESTAR EN EXPERIENCIA AUDIOLIBRO PARA PERSONAS MAYORES APLICACIÓN PREVIA Y POSTERIOR A LA EXPERIENCIA

Fecha de aplicación:____

Instrucción

Este cuestionario breve tiene como finalidad conocer sobre su bienestar en dos etapas, previo y posterior al uso de la aplicación audiolibro.

Es importante tener en consideración las siguientes definiciones:

Se entenderá por **"recurso tecnológico"** a los elementos como celular, computador, tablet y también, aplicaciones móviles.

Se entenderá por **"aplicación móvil"** una aplicación que se encuentra en el teléfono inteligente para desarrollar una función. Ejemplo: "comunicarse con otras personas" Whatssapp, "pedidos de comida" Pedidos Ya, "ver videos" YouTube.

SERVICIO DE SALUD CONCEPCION
COMITÉ ÉTICO CIENTÍFICO
04 MAYO 2021
CONCEPCIÓN

Frente a cada pregunta usted deberá marcar con una X para responder si se encuentra totalmente en desacuerdo, en desacuerdo, ni de acuerdo ni en desacuerdo, de acuerdo o totalmente de acuerdo con el enunciado.

Parte A: CONOCER EL BIENESTAR PREVIO A LA EXPERIENCIA DE AUDIOLIBRO

Pregunta 1

¿Tengo interés en aprender a usar distintos recursos tecnológicos?

Totalmente en desacuerdo	En desacuerdo	Ni de acuerdo ni en desacuerdo	De acuerdo	Totalmente de acuerdo

Pregunta 2:

¿Siento que estoy interesado en aplicar los recursos tecnológicos a mi vida?

Totalmente en desacuerdo	En desacuerdo	Ni de acuerdo ni en desacuerdo	De acuerdo	Totalmente de acuerdo

Pregunta 3

¿Tengo una percepción positiva del uso de recursos tecnológicos?

Totalmente en desacuerdo	En desacuerdo	Ni de acuerdo ni en desacuerdo	De acuerdo	Totalmente de acuerdo

Pregunta 4

¿Siento que tengo las mismas capacidades que personas de mi edad en el uso de recursos tecnológicos?

Totalmente en desacuerdo	En desacuerdo	Ni de acuerdo ni en desacuerdo	De acuerdo	Totalmente de acuerdo

SERVICIO DE SALUD CONCEPCIÓN
COMITÉ ÉTICO CIENTÍFICO
04 MAYO 2021
CONCEPCIÓN

Pregunta 5

¿Me siento satisfecho con mi manera de usar los recursos tecnológicos?

Totalmente en desacuerdo	En desacuerdo	Ni de acuerdo ni en desacuerdo	De acuerdo	Totalmente de acuerdo

Pregunta 6

¿Siento que tengo suficiente acceso a la literatura?

Totalmente en desacuerdo	En desacuerdo	Ni de acuerdo ni en desacuerdo	De acuerdo	Totalmente de acuerdo

Parte B: CONOCER BIENESTAR PREVIO A LA EXPERIENCIA DE AUDIOLIBRO

Pregunta 1

¿Tengo interés en aprender a usar distintos recursos tecnológicos?

Totalmente en desacuerdo	En desacuerdo	Ni de acuerdo ni en desacuerdo	De acuerdo	Totalmente de acuerdo

Pregunta 2

¿Siento que estoy interesado en aplicar los recursos tecnológicos a mi vida?

Totalmente en desacuerdo	En desacuerdo	Ni de acuerdo ni en desacuerdo	De acuerdo	Totalmente de acuerdo

SERVICIO DE SALUD CONCEPCIÓN
COMITÉ ÉTICO CIENTÍFICO
04 MAYO 2021

Pregunta 3

¿Tengo una percepción positiva del uso de recursos tecnológicos?

Totalmente en desacuerdo	En desacuerdo	Ni de acuerdo ni en desacuerdo	De acuerdo	Totalmente de acuerdo

Pregunta 4

¿Siento que tengo las mismas capacidades que mis pares en el uso de recursos tecnológicos?

Totalmente en desacuerdo	En desacuerdo	Ni de acuerdo ni en desacuerdo	De acuerdo	Totalmente de acuerdo

Pregunta 5

¿Me siento satisfecho con mi manera de usar los recursos tecnológicos?

Totalmente en desacuerdo	En desacuerdo	Ni de acuerdo ni en desacuerdo	De acuerdo	Totalmente de acuerdo

Pregunta 6

¿Siento que tengo suficiente acceso a la literatura?

Totalmente en desacuerdo	En desacuerdo	Ni de acuerdo ni en desacuerdo	De acuerdo	Totalmente de acuerdo

SERVICIO DE SALUD CONCEPCIÓN
COMITÉ ÉTICO CIENTÍFICO
04 MAYO 2021
CONCEPCIÓN

Pregunta 7

¿Me siento satisfecho con la experiencia de audiolibro?

Totalmente en desacuerdo	En desacuerdo	Ni de acuerdo ni en desacuerdo	De acuerdo	Totalmente de acuerdo

Pregunta 8

¿Siento que el uso de audiolibro fue significativo para mí?

Totalmente en desacuerdo	En desacuerdo	Ni de acuerdo ni en desacuerdo	De acuerdo	Totalmente de acuerdo

Pregunta 9

¿Siento que disfrute la experiencia de audiolibro?

Totalmente en desacuerdo	En desacuerdo	Ni de acuerdo ni en desacuerdo	De acuerdo	Totalmente de acuerdo

Pregunta 10

Después del uso de audiolibro ¿me siento interesado en utilizar otras aplicaciones móviles?

Totalmente en desacuerdo	En desacuerdo	Ni de acuerdo ni en desacuerdo	De acuerdo	Totalmente de acuerdo

Pregunta 11

¿Siento que la experiencia de audiolibro me permitió un mayor acceso a la literatura?*

Totalmente en desacuerdo	En desacuerdo	Ni de acuerdo ni en desacuerdo	De acuerdo	Totalmente de acuerdo

Pregunta 12

¿Siento que la experiencia de audiolibro me facilita tener nuevos temas de conversación?

Totalmente en desacuerdo	En desacuerdo	Ni de acuerdo ni en desacuerdo	De acuerdo	Totalmente de acuerdo

¡Muchas gracias por su colaboración!

SERVICIO DE SALUD CONCEPCION
COMITE ÉTICO CIENTÍFICO
04 MAYO 2021
CONCEPCION

Anexo 3
Cuestionario para medir Usabilidad

Selecciona el grado de "acuerdo" o "desacuerdo" de cada uno de los siguientes ítems. Donde:

1: Totalmente en desacuerdo
2: En desacuerdo
3: Dudoso
4: De acuerdo
5: Totalmente de acuerdo

**Ítem**	**1**	**2**	**3**	**4**	**5**
1.- Creo que me gustaría utilizar la aplicación con frecuencia.					
2.- Me pareció que la aplicación era innecesariamente compleja.					
3.- Creo que la aplicación era fácil de usar.					
4.- Creo que voy a necesitar apoyo de una persona para usar la aplicación.					
5.- Me pareció que las funciones de la aplicación fueron bien integradas.					
6.- Pensé que había demasiada inconsistencia en la aplicación.					
7.- Creo que la mayoría aprendería a usar la aplicación rápidamente.					
8.- Me pareció que la aplicación era muy complicada de usar.					
9.- Me sentí muy seguro al usar la aplicación.					
10.- Sentí que tenía que aprender muchas cosas antes de que pudiera ponerme en marcha con la aplicación.					

SERVICIO DE SALUD CONCEPCIÓN
COMITÉ ÉTICO CIENTÍFICO
04 MAYO 2021
CONCEPCIÓN

Anexo 4

Cuestionario para medir Competencias Digitales

Marca con "X" aquellas actividades que puedes realizar por medio del uso de tecnología.

Información	Búsqueda de información sobre bienes y servicios.	
	Obtención de información de sitios web de las autoridades públicas.	
	Leer o descargar noticias/diarios/revistas en línea.	
	Copiar o mover un archivo o carpeta.	
Comunicación	Enviar/Recibir correos electrónicos.	
	Realizar llamadas de teléfono/video a través de Internet.	
	Postear mensajes en sitios de chat/Redes sociales.	
	Subir información a un sitio web para ser compartida.	
Creación de contenido	Usar o mover información en un documento.	
	Usar fórmulas básicas para sumar, restar, multiplicar o dividir en una hoja de cálculo.	
	Crear presentaciones electrónicas incluyendo por ejemplo imágenes, sonidos, videos o gráficos.	
	Crear sitios web o blogs.	
	Escribir un programa informático utilizando un lenguaje de programación especializado.	
Resolver problemas	Conectar e instalar nuevo dispositivos al computador.	
	Instalar/reemplazar un sistema operativo.	
	Modificar o verificar la configuración de un software o programa.	
	Acceder al banco a través de Internet.	

SERVICIO DE SALUD CO
COMITÉ
ÉTICO
CIENTÍFICO
04 MAYO 2021
CONCEPCIÓ

	Comprar bienes o servicios a través de Internet.	
	Vender a través de Internet.	
	Hacer una cita médica a través de un sitio web.	

SERVICIO DE SALUD CONCEPCIÓN
COMITÉ ÉTICO CIENTÍFICO
04 MAYO 2021
CONCEPCIÓN

Anexo 5

Infografía para la difusión en CCR

AUDIOLIBRO: BIENESTAR EN EL ADULTO MAYOR

- SI TIENES 60 AÑOS DE EDAD O MÁS.
- PERTENCES AL CCR CONCEPCIÓN.
- POSEES UN TELÉFONO CELULAR CON ACCESO A INTERNET O WIFI.
- Y ACTUALMENTE NO TE ENCUENTRAS PARTICIPANDO EN NINGÚN PROGRAMA
  (ACTIVIDADES EN EL CESFAM DONDE TE REÚNAS CON OTROS ADULTOS SEMANALMENTE).

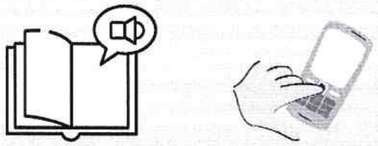

PARTICIPA EN LA EXPERIENCIA DE AUDIOLIBRO PARA EL ADULTO MAYOR

Un equipo de la U. de Concepción y de la U. Católica de la Santísima Concepción han desarrollado una aplicación para el teléfono celular, que permitirá escuchar Audiolibros de poemas de forma gratuita. Es una actividad que forma parte de una investigación. Eso significa que se harán encuestas antes y después de la experiencia de escuchar los Audiolibros. Éstas servirán, de forma anónima (sin exponer los datos de los y las participantes) para saber si esta aplicación favorece el bienestar de las y los adultos mayores.

EXPERIENCIA VOLUNTARIA Y GRATUITA

PARA INSCRIBIRTE:

- DEJA TU NOMBRE COMPLETO Y TELÉFONO PARA CONTACTARTE.

- ¿DÓNDE? EN LA SECRETARÍA DEL CCR.

DATOS DE CONTACTO DE LOS INVESTIGADORES
FLGA. VALERIA ESPEJO V.
OFICINA 3er. PISO FACULTAD DE MEDICINA UDEC
ANEXO: 412204792

SERVICIO DE SALUD CONCEPCIÓN
COMITÉ ÉTICO CIENTÍFICO
04 MAYO 2021
CONCEPCIÓN
