# Supplementary material for: How does a poetry audiobook app improve the perception of well-being in older adults? A study protocol
Source: PLoS One. 2024 Oct 31;19(10):e0312463. doi: 10.1371/journal.pone.0312463 (PMC11527330; doi:10.1371/journal.pone.0312463)

Research Project Protocol

I. GENERAL DATA

**Project title**	The use of an audiobook application in older adults belonging to the Community Rehabilitation Center and the impact on their well-being.
**Responsible Researcher(s)**	Valeria Espejo Videla
**Co-investigator(s)**	Laura Aravena Canese<br>Pedro Rossel Cid
**Sponsoring Institution**	Universidad de Concepción and Universidad Católica de la Santísima Concepción
**Source of financing or self-financing**	Self-financed

II. PROJECT DESCRIPTION

1. Justification of the Proposal and State of the Art

1.1 Older Adults, Health, and Well-being

The population of older adults has been increasing worldwide, so they have become direct recipients of public policies that favor their well-being in all areas of their lives, social, health, technological, and cultural, among others. Inequities in any of these areas should not continue to increase if we wish to improve their quality of life. This is the case of using technologies and how they favor active aging and the overall well-being of the older adult, or how, in their absence, even greater gaps are produced in the possibilities of enjoying life (Matas-Terrón et al., 2016).

During the process of older adulthood, it is important to maintain interest and active participation in sociocultural opportunities that may arise. This permanent, continuous, and daily activity aims to prevent social and family isolation and maintain independence (Villafuerte et al., 2017). Common activities such as listening to the radio, music, or audiobooks allow enjoyment, entertainment, cognitive stimulation, and favor relaxation and forgetfulness of worries (Forsblom et al., 2010).

Currently, health has a comprehensive approach. In 1948, the World Health Organization defined it as "complete physical, mental and social well-being and not merely the absence of disease or infirmity." This vision is relevant to older adults since it considers not only biomedical aspects but also the factors that affect their health (Gallardo-Peralta & Sanchez). (Gallardo-Peralta & Sánchez-Moreno, 2019; Khosravi et al., 2016).

One of the goals of health work is to seek the well-being and improve the quality of life of the elderly. The World Health Organization (WHO) states that health policies should be oriented to promote healthy aging, understood as "the process of promoting and maintaining the functional capacity that enables a person to achieve well-being in old age" (World Health Organization (WHO), 2006).

Aging involves physical, social, and cognitive changes, being a physiological state and not a synonym of disease. Therefore, aging is a biocultural state related to temporal, social, and environmental spheres (Martínez Fuentes & Fernández Díaz, 2008). This stage of the life cycle tends to be associated with a process of loss and a period of crisis, which causes some older adults to have difficulties in adapting to this phase and therefore become more vulnerable, so it is of great importance to generate comprehensive health actions to support the elderly and thus achieve a permanent development aimed at improving their well-being, so that they can adopt new roles in various contexts and enhance psychosocial and spiritual growth (Espinoza L., 2016).

Thus, well-being is part of health in its most general sense and is manifested in all areas of human activity. When an individual feels well, he/she is more productive, sociable, creative, has a positive projection of the future, instills happiness and this implies the capacity to love, work, relate socially and control the environment. The association between some emotional states and the responses to stress of one type or another has been demonstrated. (García & González, 2000) cited by Abello et al., (2008).

According to Diener (1994), cited in Zubieta & Delfino (2010), well-being can be characterized by three elements: its subjective character, which rests on the person's own experience; its global dimension, which includes the valuation of the subject in all areas of his or her life; and positive appreciation, since its nature goes beyond the mere absence of negative factors.

Ryan and Deci (2001), cited in Ortiz Arriagada & Castro Salas (2009), have proposed organizing the different studies of well-being into two major traditions: one fundamentally related to happiness, the hedonic tradition, and the other linked to the development of human potential and the realization of what one can be, defined as the eudaimonic tradition.

Hedonic or subjective well-being is understood as "the category that a person uses to judge his or her life in a general or global way" (Diener & Suh, 2000). Diener & Suh (2000) argue that Subjective Well-being is composed of the following 3 dimensions: Satisfaction with Life, Positive Affect, and Negative Affect.

Carol Ryff refers to eudaimonic or psychological well-being as the effort to perfect one's potential, it is the way in which life acquires meaning for oneself, with certain efforts to improve oneself and achieve valuable goals; the central task of people in their lives is to recognize and realize all their talents to the maximum (Keyes et al., 2002). To explain psychological well-being, Ryff formulates a multidimensional model of personal development in which he specifies six dimensions: self-acceptance, positive relationships with other people, autonomy, mastery of the environment, purpose in life, and personal growth (Rodríguez, Negrón, Maldonado, and Rodríguez, 2002).

Social well-being is the valuation of the circumstances and functioning within the society (Keyes, 1998), cited by (Blanco & Díaz, 2005). It is composed of the following dimensions: social integration, social acceptance, social contribution, social actualization, and social coherence.

1.2 Older adults and the use of technology

Older adults should be encouraged to take a positive approach to technology, promoting interest in digital literacy to strengthen the use of different digital resources, focusing on reducing fear of use, and enhancing skills based on their interests and tastes (Matas-Terrón et al., 2016a). From this perspective, access to and availability of literature through digital technologies could improve the well-being of the elderly.

The older adult population is distanced from the use of information technologies, particularly mobile applications, due to various barriers, such as physical, acceptance, and design barriers (Fletcher & Jensen, 2016). This gap in the use of digital technologies deprives them of services that could positively impact their health, such as cultural content, books, stories, or poems that are accessible on virtual platforms.

Mobile applications based on e-Health are technological resources that function as information and support delivery tools to promote health, prevent diseases, monitor diagnoses and treatments, and help control different users' lifestyles (Vukovic et al., 2018).

The use of information and communication technologies (ICTs) generates benefits in the quality of life of older people and impacts different areas, including learning, communication, daily activities, entertainment, hobbies, and health (Casamayou & Morales González, 2018). The study by Kim et al., 2017 examined how ICTs improve older adults' psychological well-being by facilitating their social connectedness. In addition, favoring instances that promote health through usable applications for the older adult would improve the perception of quality of life and increase the person's happiness. The improvement effect found was moderated by age and frailty status and was observed to reduce social isolation in this population (Fang et al., 2018).

An application that allows to connect with literature is the audiobook, which corresponds to the recording of a literary work read or dramatized by one or more people in which music or effects can be incorporated (García-Rodríguez & Gómez-Díaz, 2019). In the beginning, it was oriented to people with visual impairment or for people seeking to learn another language. Currently, it is aimed at the entire population throughout their life cycle (García-Rodríguez & Gómez-Díaz, 2019). Audiobooks are a resource that could reach a greater number of the older adult population, overcoming barriers such as illiteracy, difficulties of aging such as presbyopia, and

motor impairments, among others (Ameri et al., 2017). Audiobooks generate a positive experience and can improve the meaning of life, including physical and mental well-being (Ferrari, 2012).

While there has been research related to e-books and audiobooks, it has been mostly in child and adolescent populations (Larson, 2015; Moyer, 2012). According to the above and the review conducted, there is an opportunity for research and to make a contribution to the population of older adults, considering the use of audio of poems so that they can listen to them since there is almost no related research and it has been reported that for older adults it can be a useful tool (Macik et al., 2017; Smallfield & Kaldenberg, 2020).

Alstergren et al. developed "DreamScape," an application that allows you to create, share, and listen to stories. It was developed to be run both in a browser and on mobile devices. The main idea of this application is that users can create stories by selecting different options as the story progresses (multiple choice), stories that can eventually have different endings. This application was evaluated by 4 people whose ages were between 22 and 60 years old. It is worth mentioning that this application is more focused on creating stories, as a game element that allows people to improve their well-being. The element that allows listening to the story is complementary to the above.

Endrstova et al. developed a physical unit for the playback of audio content tailored to visually impaired older adults' specific needs and preferences. In particular, this unit was developed with familiar metaphors for users in mind, such as turning on and off the reading by opening and closing the book accordingly. It also has functions such as volume control, skipping chapters, rewinding, bookmarking, and switching between particular book titles that can be presented by the device. Several evaluations were conducted, one of them with 7 visually impaired older adults with an average age of 73 years, living in a residence for older adults. The evaluation lasted half an hour.

Poerio & Totterdell, conducted a longitudinal randomized controlled trial, where audiobooks' effect on older adults' well-being was studied. The participants were 94 older adults who used an MP3 player for a period of six weeks. This contained the books to be listened to. The study showed that the subjects who reported greater absorption and appreciation of the audiobook also reported greater post-intervention well-being, extending until after the book's end. According to the authors, using an audiobook can have a lasting positive impact on several aspects of well-being in older adults.

On the other hand, it is important that e-health-oriented tools are interactive, interoperable, easy to use, motivating, adaptable, and accessible to different recipients (Kreps & Neuhauser, 2010). Thus, knowing the usability of an application is essential to determine whether it is efficient and practical in its use.

Usability is defined as the extent to which a system, product, or service can be used by users to achieve specific objectives with effectiveness, efficiency, and satisfaction in each context of use (ISO 9241-11, 2018). According to Rubin & Chisnell, for a product to be usable, it must be useful, efficient, effective, satisfying, easy to learn, and accessible. The same authors indicate what each of these properties refers to:

- Utility: The degree to which a product enables the user to achieve its objectives, and it is an assessment of the user's willingness to use the product.

- Efficiency: The speed with which the user's objective can be achieved accurately and completely, usually a measure of time.
- Effectiveness: The extent to which the product behaves the way users expect it to and the ease with which users can use it to do what they want. It is usually measured quantitatively with the error rate.
- Satisfaction: The user's perceptions, feelings, and opinions about the product, usually captured through written and oral questions.
- Ease of Learning: This is part of effectiveness and concerns the user's ability to operate the system at a defined level of proficiency after a predetermined amount and period of training (which may not take time). It may also refer to the ability of infrequent users to relearn the system after periods of inactivity.
- Accessibility: In a broad sense, accessibility is about having access to the products needed to achieve a goal. In a narrower sense, making products usable by people with disabilities.

Evaluating usability is one of the most important tasks when building a software tool's user interface, which will become the visible part of the interaction with the application and probably the one with which errors will occur in such interaction. The idea of an evaluation is to get feedback from the users to identify the objectives achieved or monitor the use of the product. Therefore, different characteristics that may affect the normal operation of the application, such as interactivity, ease of use, and, above all, aesthetics, must be taken into account. (Zhang & Adipat, 2005).

1.3 Presentation of the problem

Through the following study, we want to know the impact of an audiobook mobile application on the well-being of older adults and the usability of the mobile application designed for this population.

1.3.1 Justification of the problem

In Chile in recent times, there has been an accelerated increase in the elderly population, and they constitute 16.2% of the general population (Albala, 2020). (Albala, 2020). The elderly must adapt to the changes brought about by aging, which makes them vulnerable from different aspects, including economic, access to technology, education, and health.

In the field of health, declines can be evidenced at the visual, cognitive, and functional levels; in relation to visual deficits, they tend to interfere with activities of daily living and at the psychological level, generating loss of self-esteem and social isolation, among others (Loh & Ogle, 2004). With respect to functionality in this population, it is difficult for them to perform basic or instrumental daily activities (Mora Quezada et al., 2004).

The role of reading in older populations has been studied and it has been determined that pleasurable reading is associated with benefits in health and well-being (Poerio & Totterdell, 2020). Another alternative that older adults have is listening to audiobooks, which significantly affects well-being and meaning in life (Poerio & Totterdell, 2020).

Because there is a massification in the use of technologies, there is an increase in digital consumers and we are in a digital culture (Guaña-Moya et al., 2017), therefore, it becomes relevant to empower the older population so that they have greater access to free technological instances that allow them to connect with such powerful areas in an individual inserted in community, such as literature and culture.

1.3.2 Impact

The relevance of determining whether the use of audiobooks has an impact on the well-being of the elderly is that it is linked to a health benefit from a biopsychosocial concept. Older people are isolated from the use of technology, so technological tools should be created or adapted to this particular population since they have specific needs and limitations and thus enhance the ease of use of mobile applications and satisfaction with them or other digital systems. By designing digital platforms specifically oriented to the elderly, they are considered as members of society, and at the same time, the technological gaps of this population are reduced, and they are motivated to be part of the digital community.

1.3.3 Novelty

At the time of the literature review, no studies were found in Chile on the use of audiobooks in the older population, even more, that analyze the impact it has on well-being, although there are similar studies in other countries and for the population of children and adolescents (Larson, 2015; Moyer, 2012; García-Herrera et al., 2020; Ronquillo & Peña, 2017). The use of technology in older adults through a mobile audiobook application seeks to enhance a positive approach to technology, promoting interest in digital literacy to strengthen the use of different digital resources by focusing on reducing the fear of use and enhancing the skills based on their interests and tastes (Matas-Terrón et al., 2016a).

The idea is that older adults integrate technology as a resource that allows them to access and enjoy their interests, therefore a mobile application designed according to their characteristics is proposed. The above opens a door and brings them closer to social spaces, such as access to literature and culture.

As already discussed, audiobooks generate a positive experience and can enhance the meaning of life including aspects of both physical and mental well-being (Poerio & Totterdell, 2020).

2. Research Objectives/Hypothesis

2.1 Research question

Does listening to poetry audios using a mobile application improve the well-being perception of older adults attending the Community Rehabilitation Center (CRC) of Concepción?

2.2 Hypotheses

H1: The use of a mobile application that allows listening to audio of poems produces a positive change in the perception of well-being in older adults.
H0: The use of a mobile application that allows listening to audio of poems does not produce a positive change in the perception of well-being in older adults.

2.3 Objectives

2.3.1 General Objective

To determine the changes in the perception of the well-being of older adults who receive care at CRC Concepción, following the use of a mobile application to listen to audio of poems.

2.3.2 Specific Objectives

1. Modify the language used in the well-being questionnaire to make it understandable and appropriate for older adults.
2. Characterize the older adult population served at the CRC of Concepción.
3. Determine changes in the perception of hedonic well-being in older adults attending CRC before and after the use of the audiobook mobile application.
4. Determine changes in the perception of eudaimonic well-being of older adults seen at CRC before and after using the audiobook mobile application.
5. Determine changes in the perception of social well-being of older adults seen at CRC before and after using the audiobook mobile application.

3. Material and Method

3.1 Methodology: Qualitative/Quantitative/Mixed

Quantitative study methodology, since our purpose is to answer the research question, test our hypothesis, and comply with the general and specific objectives set forth (Hernández et al., 2014)

3.2 Study design

The study's research design corresponds to a quasi-experimental study (Hernández et al., 2014).
It is quasi-experimental since there is a deliberate intervention, i.e., the subjects are not randomly assigned, and the group is formed before the experiment. The design corresponds to a pre-test/post-test with a single group; this is measured prior to the use of the audiobook then the older adults use

the mobile application for a month, and then a final evaluation is applied. In addition, there is an initial baseline to see the group's level in the dependent variable before the stimulus.

It is descriptive because it will seek to describe whether there is a change in the perception of well-being of older adults belonging to the CRC Concepción, after using a mobile audiobook application. The variables perception of well-being and usability will be measured quantitatively to contrast the above hypothesis.

3.3 Variables

The study seeks to know the demographic characteristics, the well-being of the person pre and post-use of the mobile application, and its usability. The variables to be considered in this study are:

1. Sociodemographic characteristics
2. Hedonic well-being
3. Eudaimonic well-being
4. Social well-being
5. Audiobook application for listening to poems designed for the elderly.

The variables are detailed below at a conceptual level.

1. Sociodemographic characteristics shall be understood as:
    a. Age: Years completed since the date registered in the identity card.
    b. Sex: Male or female according to own definition.
    c. Schooling: Years of formal schooling completed by the individual.
    d. Digital competencies.

Well-being can be characterized based on three domains: hedonic, eudaimonic and social well-being.

1. Hedonic or subjective well-being is understood as the "category that a person uses to judge his or her life in a general or global way" (Diener & Suh, 2000).

2. Eudaimonic or psychological well-being as the effort to perfect one's own potential, is the way in which life acquires meaning for oneself, with certain efforts to improve oneself and achieve valuable goals; the central task of people in their lives is to recognize and realize the maximum all their talents (Romero Carrasco et al., 2007).

3. Social well-being is the valuation made of the circumstances and functioning within society (Blanco & Díaz, 2005)

4. Audiobook application for listening to poems designed for the elderly.

The audiobook corresponds to the recording of a literary work read or dramatized by one or more people in which music or effects can be incorporated (García-Rodríguez & GómezDíaz, 2019). The audiobook application is a mobile application, built especially for this study. It runs on the

Android 5.0 operating system or higher for smartphones. It is built for older adults, and the limitations that older adults have were considered in its design, according to a specific guide for this type of user (Silva et al., 2015).

It consists of a very simple interface, which is divided into 3 parts:

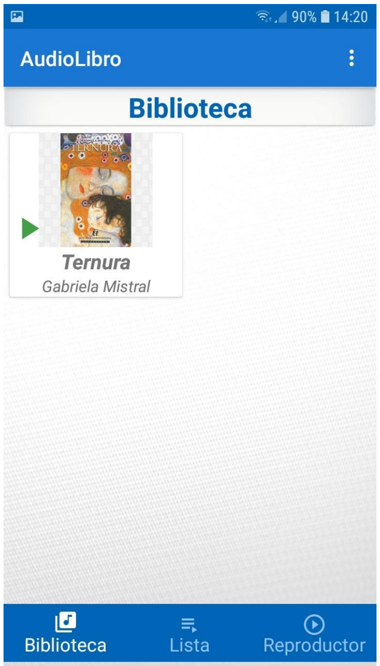

Figure 1: Library

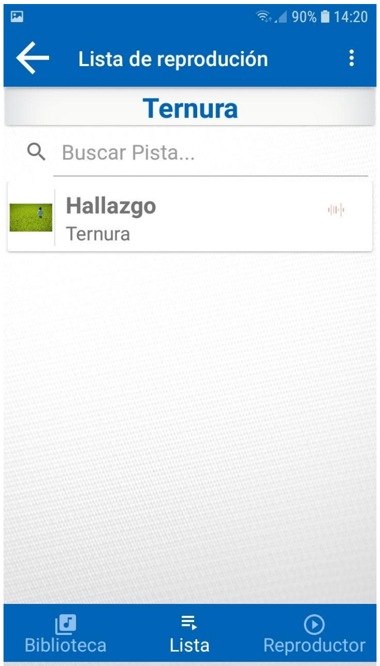

Figure 2: Playlist

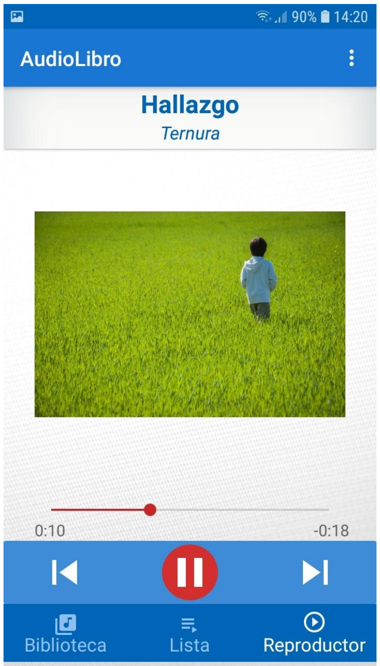

Figure 3: Player

In Figure 1 you can select the book you want to play. In Figure 2, considering the selected book, you can choose the poem (in this case) you want to listen to from all those available. Finally, in Figure 3 you can stop or replay the selected poem.

This application will automatically collect, without the intervention of the senior, information on its use, and such information will be securely stored in a server external to the application, respecting the confidentiality of the user's data by using a code to represent each of the participants. Such information will be as follows:

- identification of the older adult,
- ▪ date, time and duration of each playback,
- tracks (poems) listened to.

With respect to this information, usability (how user-friendly the application is) and frequency of use (how much the application is used daily) will be measured as independent variables.

The variables at the operational level are as follows

- The following is an operational definition of the variables of this research, which clarifies the concepts to be addressed.

1. **Sociodemographic characteristics**:
    a. Age: Independent variable, ratio scale. Subsequent analysis may categorize it into ranges.
    b. Sex: Nominal dichotomous independent variable: female/male.
    c. Schooling: Independent variable, ratio scale. Years of formal education completed. In subsequent analysis, it is possible to categorize it into: illiterate, incomplete basic education, complete basic education, incomplete secondary education, complete secondary education, and complete and incomplete higher education.
    d. Digital competencies: Independent variable, ordinal (absence, presence). Digital competence is the set of knowledge, skills, and attitudes required when using ICTs and digital media to perform tasks, solve problems, communicate, and manage information, and construct knowledge efficiently, appropriately, and autonomously. This competence can be measured through a questionnaire (Annex 4). It consists of 20 items where it is necessary to indicate whether or not the indicated activity can be developed.

2. **Eudaimonic well-being:** Ordinal dependent variable, understood as totally disagree, disagree, neither agree nor disagree, agree, totally agree.
3. **Hedonic well-being:** Ordinal dependent variable, understood as totally disagree, disagree, neither agree nor disagree, agree, totally agree.
4. **Social well-being:** Ordinal dependent variable, understood as totally disagree, disagree, neither agree nor disagree, agree, totally agree.

After exploring its validity, all the well-being variables will be processed in further analysis as a single well-being scale (ratio scale).

In the initial part, the score range is from 0 to 30 points Part A.

In the final application, the score ranges from 0 to 60 points Part A + B.

The range of scores is different because part B is added, which measures specific changes in the acceptance of the technology in everyday life after the application is used.

5. **Mobile audiobook application:** Independent variable.
    a. **Usability:** Independent variable, ratio scale score (range 0 to 100 points). Usability is, in simple terms, the ease with which users can use software. This ease can be measured with the System Usability Scale or SUS questionnaire (annex 3 for questions (Likert scale)). This test consists of 10 items with five response options for respondents, ranging from "strongly disagree" to "strongly agree."
    b. **Frequency of use:** Independent variable, ratio scale. Number of audios listened to per day.

3.4 Population and sample

Population from which the participants will come:

The population corresponds to older adults (MA) aged 60 years and older who receive primary care at the Community Rehabilitation Center in the commune of Concepción. The municipality of Concepción has 223,574 inhabitants, of which 12.46%, corresponding to 27,858 are older adults (National Institute of Statistics, 2017).

The Community Rehabilitation Center (CRC) Concepción serves people with GES pathologies of neurological and non-neurological origin. The care is based on community-based rehabilitation, so individual and group activities are carried out, as well as education in health promotion and disease prevention for the community, home visits, specific intervention by professionals such as Physical Therapist and Occupational Therapist of the center, training center in teaching assistance agreement with the career of Speech Therapy, Occupational Therapy, Physical Therapist, and networking. It currently serves users who are enrolled in the CESFAM O'Higgins, Juan Soto Fernandez, and Tucapel. In 2019, 250 older adults were admitted to CRC, and in 2018 they were 320; the above data were provided by the Manager of CRC Concepción, physical therapist Nadia Muñoz.

Participant selection criteria: Inclusion-Exclusion

Inclusion Criteria:

- People aged 60 or over who belong to the CRC Concepción.
- People volunteer to participate in the study and confirm it by signing the informed consent form.
- Skilled and able to approve informed consent.
- Who owns a smartphone where to run the audiobook mobile application.
- Older adults who are not participating in another program, either emotionally, socially, and/or motorically.

Exclusion Criteria

- Significant psychiatric or medical illness (depressive disorder, delirium, intellectual disability, etc.).
- Presence of neurodegenerative diseases (dementia, Alzheimer's disease, Parkinson's disease, multiple sclerosis, etc.).
- Older adults classified with dependency criteria.
- Presence of a severe sensory deficit, either in visual or hearing impairment.
- Illiterate people.

Sample size calculation

The type of sampling is non-probabilistic by convenience; the mode of recruitment of participants is explained below. Briefly, we will invite people treated at CRC who meet the inclusion and exclusion criteria and wish to participate through an open call. A maximum of 60 participants has been estimated considering the study by Ameri et al., (2017), in which the sample was 60 people who voluntarily agreed to participate. On the other hand, in a study by Poerio and Totterdell (2020), the sample size was 90 participants who were invited to participate voluntarily. Against this background, the number set as a maximum seems appropriate. With respect to the minimum necessary to carry out the pre and post-use evaluation of the app, a minimum of 30

participants has been considered to obtain data from the different quintiles and genders within the sample.

Description of the participant recruitment process

After authorization from the head of the DASM of Concepción and the administrative head of the CRC Concepción, recruitment will be carried out as follows:

Posters will be placed on the mural of the CRC to make an open call to all people over 60 who attend this place and wish to participate in the research. The poster will have written on it the place where they must go within the center’s premises to register. When applying for registration, you will be asked for your full name and telephone number so that you can be contacted.

In addition, the project managers will schedule group meetings at the CRC's facilities to invite participation in the research.

Once the interested parties have registered, the subjects will be contacted by telephone to invite them to participate voluntarily. In this instance, information will be provided clearly and concisely, indicating the research objectives and the strategy to be used. In this instance, key questions will be asked to determine if they meet the criteria for inclusion in the study and participation will be confirmed.

If the user refuses to participate, they will be thanked, and there will be no harm to them.

In case of acceptance, people will be invited to the CRC at a time agreed upon with them and the informed consent will be explained verbally with the support of the written document (see informed consent in Annex 1), emphasizing that this is a voluntary study in which the confidentiality of your personal information will be safeguarded and ensured.

If they agree, the document will be signed (a copy will be given to the participant), and the application of the surveys and questionnaires prior to the use of the application will continue immediately.

At the CRC office, one of the project managers will be in charge of explaining how to answer the questionnaires, which are self-administered. The user will be accompanied while answering the questionnaires in case any doubt arises. Digital skills questionnaire and pre-experience questions will be applied to the questionnaire on well-being in audiobook experience for older adults.

Subsequently, the operation of the audiobook application will be explained, and a contact phone number will be provided to resolve any queries that may arise. Two weeks after the delivery of the application, they will be contacted to monitor the process. After one month of use, they will be invited to complete the well-being and usability questionnaires.

After the evaluation is completed, the data analysis will be carried out.

3.5 Data collection instrument

It is important to note that the measurement will be applied in a box of the health device, with good lighting, availability of chairs and without distractions, at the time previously agreed upon in the contact telephone call. The researchers will be the ones who will carry out the evaluations before and after the use of the application.

The information collection instruments are presented below.

Questionnaire on Well-being in the audiobook experience for the elderly

Well-being will be assessed through a questionnaire on well-being in the audiobook experience for older adults, created by the researchers for this study. It seeks to learn about well-being in older adults before and after using the mobile application. It presents a stage A containing 6 questions, which is applied before the use of the application, and a part B, which is composed of 12 questions and is applied after the use of the mobile application (Annex 2). The questions are all on a Likert scale from Strongly Disagree (0) to Strongly Agree (5), specifying a minimum score for Part A of 0 points to a maximum of 30 points and for Part B from a minimum of 0 points to a maximum of 60 points.

To adjust the language of the questionnaire, a small group of 5 to 10 older adults will be selected to review how the instrument is written and provide feedback. The idea is that if there are concepts or wording they do not understand or if the words used are not adapted to the local and group reality, these people can suggest changes that will be adopted.

Questionnaire to measure the Usability of an Application (SUS)

The *System Usability Scale* (SUS) was invented by John Brooke who, in 1986, created this "quick and simple" usability scale to evaluate virtually any type of system or application (Brooke, 1996).

The SUS questionnaire uses a Likert scale and is composed of 10 carefully selected questions. In each question, the respondent selects the degree of "agree" or "disagree" with the proposed example and evaluates this on a five-point scale, where 1 means "Strongly Disagree" and 5 means "Strongly Agree."

To calculate the SUS score, the contributions of each item must first be summed. The contribution of each item will be worth between 0 and 4.

For items 1, 3, 5, 7, and 9, the contribution will be the scale position less than 1. For items 2, 4, 6, 8, and 10, the contribution will be 5 minus the scale position. The sum of the results is multiplied by 2.5 to obtain the overall SUS value. The result will be between 0 and 100, the higher the result, the higher the level of usability of the system or application.

The questionnaire to measure the usability of an application is presented in Annex 3.

Questionnaire to measure Digital Competencies

Digital competence has been recognized as one of the European Union's 8 key competencies for lifelong learning. It can be broadly defined as the confident, critical, and creative use of ICTs to achieve goals related to work, entrepreneurship, learning, leisure, inclusion, and/or participation in society (Ferrari, 2012).

In the questionnaire for measuring digital competencies (DIGCOMP) (Ferrari, 2012) identifies four areas of relevant competencies: information, communication, content creation and

problem solving. The questionnaire is evaluated considering 3 levels: **None**, **Basic**, and **Above basic**, where:

- **None**: If the person cannot perform any of the items in the area.
- **Basic**: If the person manages to perform only one of the items in the area.
- **Basic envelope**: If the person can perform at least two of the items in the area.

Each of the areas is assigned one of these labels according to the responses. Then, to evaluate the questionnaire and obtain an overall indicator of the results, the results are evaluated as follows:

- **No**: Refers to people who answered none in all areas, in addition to those who have not used the Internet in the last 12 months or who have never used the Internet.
- **Low**: Refers to people who have one or more "none" in three areas.
- **Basic**: Refers to people who have one or more "basic", but not "none".
- **Basic envelope**: Refers to people with "over basic" in the four areas.

The questionnaire to measure digital competencies is presented in Annex 4.

Data collection process and its management

Data collection process: An initial meeting will be held in which the questionnaires to measure the well-being part A of the questionnaire and the participant's digital skills will be applied. An induction for the use of the application will be made, the audiobook application will be installed in the smartphone and the participant will be called back in a period of one month to apply the well-being survey part B and the usability questionnaire.
For the application of the instruments, each participant will be assigned a code to protect and safeguard their personal data.

Data management: The database will be confidential and managed exclusively by the researchers; each participant will be identified with a code to safeguard the confidentiality of the information.

3.6 Data analysis proposal

For the analysis of the results, a single database will be created with the tabulated results of the instruments and the sociodemographic characterization data of the participants. Subsequently, descriptive statistics will be used through univariate and bivariate frequency analysis and measures of central tendency (mean and standard deviation) to describe the characteristics of the sample and the results of the questionnaires.

In the second stage, the study hypotheses will be tested by means of a multivariate linear regression to predict the well-being variable at the end of the intervention, explained by initial well-being, and controlled by sociodemographic variables, usability, and frequency of use. We will explore whether covariates have confounding and/or modifying effects on the relationship between

initial and final well-being scores. The specific model is assumed to be a Generalized Linear Model, for normal distribution and link identity. Data processing and analysis will be performed with IBM Statistical Package for Social Sciences (SPSS) software. The budgeted statistical model can be summarized as:

$$Bfinal = a_0 + b(Binicial) + b(frec) + b(usabilidad) + b(sexo) + b(otrasdem)$$

Where:
Bfinal: final well-being score, a_0 :
intercept
Binicial: pending initial well-being
B(frec): slope associated with the frequency of app use.
B(usability): slope associated with the evaluated app usability.
B(sex): participant's sex
B (other dem): slopes associated with other confounding demographic variables or relevant modifying effects.

3.7 Ethical considerations

The present study corresponds to a research project carried out jointly by the Department of Speech Therapy of the Universidad de Concepción and the Department of Computer Science of the Universidad Católica de la Santísima Concepción and will comply with the Good Clinical Practices given by the Chilean Government. Compliance with the Patient's Rights and Duties Law (Law 20,584) will be safeguarded, and all data collection and management aspects will be included. In addition, the research to be performed is under the ethical norms of the Helsinki Declaration.

The information that will be requested from the participants corresponds to: informed consent, application of well-being questionnaires, questionnaire to measure the usability of an application (SUS), and questionnaire to measure digital competencies (DIGCOMP). The well-being questionnaire will be carried out before (part A) and after use (part B) of the mobile application, the DIGCOMP before use, and the SUS after the experience. The data collected and provided by the participants are extremely valuable and confidential, therefore, only members of the research team will have access to the information. Privacy will be protected, and for this purpose, each participant will be assigned a code to protect identity.

To safeguard the ethics in the execution of the research, the "Ethical requirements for research on human subjects" were contemplated according to the following (Rodríguez Yunta, 2004):

- Users who voluntarily agree to participate in the research. They will be asked to sign an informed consent form to evidence this process.
- Informed consent: each of the invited participants will decide their participation voluntarily; the consent will be in verbal and written form, which will detail the procedure and the implications of participation in the research, oriented in the ethical principles of the Declaration of Helsinki, will answer any questions that the participant presents and will be given a copy of the informed consent with a contact number for any concerns that may arise. Participation is voluntary, and information will be protected, i.e., every precaution will be taken to protect and respect the privacy of the participant and the confidentiality of

his/her personal information through the use of codes to refer to the participant. The research has no associated costs for the participants. The expected benefits correspond to a change or maintenance of their well-being, in addition to being a tool that brings them closer to the culture through the use of technological tools. In the social area, it is expected to be a tool that contributes to the integral health of older adults. The use of the results of the research will be relevant to demonstrate the benefits of the application in the integral health of the older adults, and to promote the use of this type of tools. In conclusion, the informed consent process will be based on the following pillars: providing clear, understandable, and detailed information, ensuring the participant's understanding, the subject's free will, and the space to answer all kinds of doubts that need to be resolved.

- Respect for the enrolled subjects: The investigators will guarantee the confidentiality of the participants and will also respect the user's right to withdraw from the study when warranted without prejudice.

With respect to the ethical implications of the project, the research team is aware that the use of the information, privacy, and confidentiality of the subject and his/her data, as it is sensitive information, and its use will be only for the objectives described and limited to what is expressed in this document. The participant will give permission through his/her signature in the informed consent form to access the information, based on laws 20,120, and 20,584.

4. Work plan

Due to the global contingency, it is impossible to detail specific actions.

Activities	Deadlines
Generate and validate survey language to be applied to older adults.	Month 1
Application development.	Month 3 to month 4
Create a Research Protocol for "Audiobook".	Month 2
Submit the research project to the CEC (Scientific Ethical Committee) of the Concepción Health Service.	Month 2 and month 3
Provide content to be used in the "Audiobook" application for older adults.	Month 3 and Month 4
Conduct the pilot test of the application.	Month 4 to month 5

Call for participants.	Month 6
Apply Questionnaires.	Month 7
Monitor the use of the “Audiobook” application in the sample.	Month 8
Apply the surveys.	Month 9
Collect “Audiobook” usage data.	Month 10
Analyze the data obtained.	Month 11
Writing a paper for publication.	Month 11 to 15
Disseminate the application free of charge.	Month 13 and 14
Maintain the Audiobook platform and subsequent updates.	Month 14 to month 24

5. Bibliography

Abello, R., Amarís, M., Blanco, A., Madariaga, C., Díaz, D., & Arciniégas, T. (2008). Well-being, self-esteem, depression and anomie in people who have not been victims of political and social violence. *Research & Development*, *16*(2).

Albala, C. (2020). THE AGING OF THE CHILEAN POPULATION AND THE CHALLENGES FOR THE HEALTH AND WELL-BEING OF THE ELDERLY. *Revista Médica Clínica Las Condes*, *31*(1). https://doi.org/10.1016/j.rmclc.2019.12.001

Alstergren, I., Andersson, A., Hedman, L., & Maric, D. (2020). *DreamScape, a Platform for Creating, Sharing and Listening to Interactive Stories*. Uppsala University.

Ameri, F., Vazifeshenas, N., & Haghparast, A. (2017). The impact of audio book on the elderly mental health. *Basic and Clinical Neuroscience*, *8*(5). https://doi.org/10.18869/NIRP.BCN.8.5.361

Blanco, A., & Díaz, D. (2005). Social well-being: Theoretical structure and measurement. *Psicothema*, *17*(4).

Brooke, J. (1996). SUS: A quick and dirty usability scale. In *Usability Evaluation In Industry* (pp. 189-194).

Casamayou, A., & Morales González, M. J. (2018). Elderly people and digital technologies: a dual challenge Idosos e tecnologias digitais: desafios duplos. *Conocimiento y Sociedad*, *7*(2), 199-226.

Diener, E., & Suh, E. M. (2000). This excerpt from Culture and Subjective Well-Being. In *books.google.com*.

Endrstova, B., Macik, M., & Treml, L. (2018). Reprobooktor: A concept of audiobook player for visually impaired older adults. *Proceedings of the 9th IEEE International Conference on Cognitive Infocommunications (CogInfoCom 2018)*, 63-68.

Fang, Y., Chau, A. K. C., Wong, A., Fung, H. H., & Woo, J. (2018). Information and communicative technology use enhances psychological well-being of older adults: the roles of age, social connectedness, and frailty status. *Aging and Mental Health*, *22*(11). https://doi.org/10.1080/13607863.2017.1358354. https://doi.org/10.1080/13607863.2017.1358354

Ferrari, A. (2012). *Digital competence in practice: an analysis of frameworks*. Technical Report, Research Centre of the European Commission.

Fletcher, J., & Jensen, R. (2016). Overcoming barriers to mobile health technology use in the aging population. *Online Journal of Nursing Informatics*, *19*(3), 1.

Gallardo-Peralta, L. P., & Sánchez-Moreno, E. (2019). Successful aging and personal well-being among the chilean indigenous and non-indigenous elderly*. *Aquichan*, *19*(3). https://doi.org/10.5294/aqui.2019.19.3.9. https://doi.org/10.5294/aqui.2019.19.3.9

García-herrera, D. G., Cárdenas-cordero, N. M., & Erazo-álvarez, J. C. (2020). *Reading comprehension and educational innovation: strategies to improve literacy in high school youth*. *VI*, 337-363. https://doi.org/10.35381/cm.v6i1.337

García-Rodríguez, A., & Gómez-Díaz, R. (2019). Reading with your ears: audiobooks and children's and young adult literature. *ThinkEPI Yearbook*, *13*. https://doi.org/10.3145/thinkepi.2019.e13c01. https://doi.org/10.3145/thinkepi.2019.e13c01

García, C., & González, I. (2000). THE PSYCHOLOGICAL WELL-BEING CATEGORY AND ITS RELATIONSHIP WITH OTHER SOCIAL CATEGORIES. *Cuban Journal of General Comprehensive Medicine*, *16*(6).

Guaña-Moya, E. J., Quinatoa-Arequipa, E., & Pérez-Fabara, M. A. (2017). Trends in the use of technologies and technological consumer behavior. *Ciencias Holguín*, *23*(2), 15-30.

Hernández, R., Fernández, C., & Baptista, P. (2014). Research methodology. In *Journal of Chemical Information and Modeling* (Vol. 53, Issue 9). https://doi.org/10.1017/CBO9781107415324.004

National Institute of Statistics (2017). *Compendio Estadístico (Statistical Compendium)*. www.ine.cl.

ISO 9241-11. (2018). *Ergonomics of human-system interaction - Part 11: Usability: Definitions and concepts.* https://www.iso.org/obp/ui/#iso:std:iso:9241:-11:ed-2:v1:en

Keyes, C. L. M., Shmotkin, D., & Ryff, C. D. (2002). Optimizing well-being: The empirical encounter of two traditions. *Journal of Personality and Social Psychology*, *82*(6). https://doi.org/10.1037/0022-3514.82.6.1007. https://doi.org/10.1037/00223514.82.6.1007.

Khosravi, P., Rezvani, A., & Wiewiora, A. (2016). The impact of technology on older adults' social isolation. *Computers in Human Behavior*, *63*, 594-603. https://doi.org/10.1016/j.chb.2016.05.092

Kim, J., Lee, H. Y., Candace Christensen, M., & Merighi, J. R. (2017). Technology access and use, and their associations with social engagement among older adults: Do women and men differ? In *Journals of Gerontology - Series B Psychological Sciences and Social Sciences* (Vol. 72, Issue 5). https://doi.org/10.1093/geronb/gbw123

Kreps, G. L., & Neuhauser, L. (2010). New directions in eHealth communication:

Opportunities and challenges. *Patient Education and Counseling*, *78*(3), 329-336.
Larson, L. C. (2015). E-Books and Audiobooks: Extending the Digital Reading Experience. *The Reading Teacher*, *69*(2), 169-177. https://doi.org/10.1002/trtr.1371
Leiton Espinoza, Z. E. (2016). Healthy aging and wellness: a challenge and an opportunity for nursing. *Enfermería Universitaria*, *13*(3). https://doi.org/10.1016/j.reu.2016.06.002
Loh, K. Y., & Ogle, J. (2004). Age related visual impairment in the elderly. In *Medical Journal of Malaysia* (Vol. 59, Issue 4).
Macik, M., Maly, I., Balata, J., & Mikovec, Z. (2017). How can ICT help the visually impaired older adults in residential care institutions: The everyday needs survey. *Proceedings of the 8th IEEE International Conference on Cognitive Infocommunications (CogInfoCom 2017)*, 157-164.
Martínez Fuentes, A. J., & Fernández Díaz, I. E. (2008). Elderly and health. *Cuban Journal of General Comprehensive Medicine*, *24*(4).
Matas-Terrón, A., Leiva Olivencia, J. J., & Franco Caballero, P. D. (2016a). FORECASTING TRAINING NEEDS FOR ACTIVE AGING. *Píxel-Bit, Revista de Medios y Educación*, *48. https://doi.org/10.12795/pixelbit.2016.i48.15.* https://doi.org/10.12795/pixelbit.2016.i48.15
Matas-Terrón, A., Leiva Olivencia, J. J., & Franco Caballero, P. D. (2016b). FORECASTING TRAINING NEEDS FOR ACTIVE AGING. *Píxel-Bit, Journal of Media and Education*, *48*, 225-240. https://doi.org/10.12795/pixelbit.2016.i48.15.
Mora Quezada, J. de las N., Osses Paredes, C. F., & Rivas Arenas, S. M. (2017). Functionality of the older adult of a Family Health Center Hualpén-Chile. *Rev. Cuba. Enferm*, *33*(1), 18-30. http://scielo.sld.cu/scielo.php?script=sci_arttext&amp%0Apid=S0864-03192017000100004%0Ahttp://www.revenfermeria.sld.cu/index.php/enf/article/view/4 32
Moyer, J. E. (2012). Audiobooks and E-books: A Literature Review. *Reference & User Services Quarterly*, *51*(4), 340-354.
World Health Organization (WHO). (2006). *Constitution of the World Health Organization. 45. Geneva. Retrieved November 2015*. 1. http://www.who.int/governance/eb/who_constitution_sp.pdf.
Ortiz Arriagada, J. B., & Castro Salas, M. (2009). The psychological well-being of the elderly and their relation with self-esteem and self-efficiency: Nursing contribution. *Cienc. Enferm*, *15*(1). https://doi.org/10.4067/S0717-95532009000100004
Poerio, G., & Totterdell, P. (2020). The Effect of Fiction on the Well-Being of Older Adults: A Longitudinal RCT Intervention Study Using Audiobooks. *Psychosocial Intervention*, *29*(1), 29-37.
Rodríguez,Y.Negrón, N. Maldonado, Y., Quiñones, & A. Osorio, N. (2015). *Dimensions of psychological well-being and perceived social support in relation to gender and university level of study*. *33*(1), 31-43. http://www.scielo.org.co/pdf/apl/v33n1/v33n1a03.pdf
Rodríguez Yunta, E. (2004). Comités De Evaluación Etica Y Científica Para La Investigación En Seres Humanos Y Las Pautas Cioms 2002. *Acta Bioethica*, *10*(1), 37-47. https://doi.org/10.4067/S1726-569X2004000100005
Romero Carrasco, A., Brustad, R., & Garcia Mas, A. (2007). Psychological well-being and its use in the psychology of exercise, physical activity and sport. *Revista Iberoamericana de Psicología Del Ejercicio y El Deporte*, *2*(2).

Ronquillo, C. C., & Peña, J. M. (2017). The audiobook as a technological tool for the learning of students of the library and archival studies at the University of Guayaquil. *Espirales: Multidisciplinary Journal of Research*, *1*(10). https://doi.org/10.31876/re.v1i10.207. https://doi.org/10.31876/re.v1i10.207

Rubin, J., & Chisnell, D. (2008). *Handbook of Usability Testing* (2nd ed.) Wiley Publishing, Inc.

Silva, P. A., Holden, K., & Jordan, P. (2015). Towards a List of Heuristics to Evaluate Smartphone Apps Targeted at Older Adults: A Study with Apps that Aim at Promoting Health and Well-Being. *Proceedings of the 48th Hawaii International Conference on System Sciences*, 3237-3246.

Smallfield, S., & Kaldenberg, J. (2020). Occupational Therapy Practice Guidelines for Older Adults With Low Vision. *The American Journal of Occupational Therapy*, *74*(2), 7402390010.

Villafuerte, J., Yenny, R., Abatt, A., Alonso, Y., Yuleydi, V., Guardado, A., & Leyva, I. (2017). Elderly well-being and quality of life , a challenge for inter-sectoral action. *Medisur*, *1*.

Vukovic, V., Favaretti, C., Ricciardi, W., & De Waure, C. (2018). HEALTH TECHNOLOGY ASSESSMENT EVIDENCE on E-HEALTH/M-HEALTH TECHNOLOGIES: EVALUATING the TRANSPARENCY and THOROUGHNESS. *International Journal of Technology Assessment in Health Care*, *34*(1). https://doi.org/10.1017/S0266462317004512. https://doi.org/10.1017/S0266462317004512.

Zhang, D., & Adipat, B. (2005). Challenges, Methodologies, and Issues in the Usability Testing of Mobile Applications. *International Journal of Human-Computer Interaction*, *18*(3), 293-308.

Zubieta, E., & Delfino, G. (2010). Life satisfaction, psychological well-being and social well-being in university students in Buenos Aires. *Anuario de Investigaciones*, *17*(1).

Annex 1

INFORMED CONSENT FOR CLINICAL RESEARCH

Title of research: The use of an audiobook application in older adults belonging to the Community Rehabilitation Center and the impact on their well-being.

You have been invited to participate in the research “The use of an audiobook application in older adults belonging to the Community Rehabilitation Center and the impact on their well-being”.

The objective of this research is to determine the changes in the perception of the well-being of older adults who receive care at CRC Concepción following the use of a mobile application to listen to poetry audios.

To decide to participate in this research it is important to consider the following information:

Participation: Your participation will consist of personally answering 2 questionnaires, using the audiobook application to listen to poems for 1 month, and answering 2 more questionnaires at the end of this period. The responsible researchers will invite you to the CRC Concepción to complete these questionnaires on a date to be agreed upon. The duration of the research will be approximately 6 weeks.

Benefits: You will not receive any direct benefit or reward for participating in this study, nor will you receive financial compensation. However, your participation will generate information to determine if there are changes in the perception of well-being in older adults after listening to poems in the audiobook application.

Risks: There is no harm to you; however, manipulating the application on your phone may increase mobile data consumption. The administration of the questionnaires, which takes approximately one hour, can cause fatigue.

Voluntariness: Your participation is absolutely voluntary. You are free to answer the questions you wish, decide not to participate, and stop your participation at any time. This will not imply any damage to you.

Confidentiality: All your opinions, responses, and data will be kept confidential and held in strict confidence. Your name will not be associated with the research at any time. All information and data will be kept only by the responsible researchers through a numerical code for the identification of each participant so that no personal data will be used.

Knowledge of the results: You have the right to know the research results. If you wish to know the global results of the research, you can request them from the research team members. In the case of obtaining any results of clinical relevance, the research team is committed to informing the participant and the treating healthcare team.

Contact information: Feel free to email the responsible researchers with any questions you may have at any time during your participation.

Flga. Valeria Espejo Videla; mail: valeriaespejo@udec.cl
Flga. Laura Aravena Canese; e-mail: laravena@udec.cl
Dr. Pedro Rossel Cid; mail: prossel@ucsc.cl
Contact: +56983375946

Address: Janequeo without number at the corner of Chacabuco. Faculty of Medicine, 3rd floor, Department of Speech, Language and Hearing Sciences.

*If you have any questions or comments about this study, please contact the Scientific Ethical Committee of the Servicio de Salud Concepción, chaired by Dr. Nelson Pérez Terán. **Email:** cec@ssconcepcion.cl. **Phone**: 56-41-2722745, red. Minsal 412745. **Address**: Hospital Guillermo Grant Benavente. San Martín 1436, Concepción. **Web Page:** http://cec.dssc.cl

INFORMED CONSENT FORM

Title of research: The use of an audiobook application in older adults belonging to the Community Rehabilitation Center and the impact on their well-being.

I,____, agree to voluntarily participate in the study entitled “The use of an audiobook application in older adults belonging to the Community Rehabilitation Center and the impact on their well-being”.

I declare that I have read and understood what my participation in this study consists of and that I may withdraw if I wish to do so.

____

Participant

Participant name: ______________________ Date: Signature:

Name Responsible Researcher: ____________ Date: Signature:

Date: Signature:

Concepción, April 2021.

Annex 2

QUESTIONNAIRE ON WELL-BEING IN AUDIOBOOK EXPERIENCE FOR OLDER ADULTS PRE- AND POST-EXPERIENCE APPLICATION

Application Date:____

Instruction

This brief questionnaire is designed to assess your well-being in two stages: before and after using the audiobook application.

It is important to take into consideration the following definitions:

“Technological resources” shall be understood as items such as cell phones, computers, tablets, and mobile applications.

Mobile application" shall mean an application that is on the smartphone to perform a function. Example: “communicate with other people” WhatsApp, “order food” Pedidos Ya, “watch videos” YouTube.

For each question, mark with an X to answer whether you totally disagree, disagree, neither agree nor disagree, agree, or totally agree with the statement.

Part A: GETTING TO KNOW YOUR WELL-BEING PRIOR TO THE AUDIOBOOK EXPERIENCE

Question 1

Am I interested in learning how to use different technological resources?

Fully in disagreement	Disagree	Neither agree nor disagree	Agreed	Totally agree

Question 2:

Do I feel that I am interested in applying technological resources to my life?

Fully in disagreement	Disagree	Neither agree nor disagree	Agreed	Totally agree

Question 3

Do I have a positive perception of the use of technological resources?

Fully in disagreement	Disagree	Neither agree nor disagree	Agreed	Totally agree

Question 4

Do I feel that I have the same capabilities as people my age in the use of technological resources?

Fully in disagreement	Disagree	Neither agree nor disagree	Agreed	Totally agree

Question 5

Am I satisfied with the way I use technology resources?

Fully in disagreement	Disagree	Neither agree nor disagree	Agreed	Totally agree

Question 6

Do I feel I have sufficient access to literature?

Fully in disagreement	Disagree	Neither agree nor disagree	Agreed	Totally agree

Part B: KNOWING WELL-BEING BEFORE THE AUDIOBOOK EXPERIENCE

Question 1

Am I interested in learning how to use different technological resources?

Fully in disagreement	Disagree	Neither agree nor disagree	Agreed	Totally agree

Question 2

Do I feel that I am interested in applying technological resources to my life?

Fully in disagreement	Disagree	Neither agree nor disagree	Agreed	Totally agree

Question 3

Do I have a positive perception of the use of technological resources?

Fully in disagreement	Disagree	Neither agree nor disagree	Agreed	Totally agree

Question 4

Do I feel I have the same capabilities as my peers in the use of technological resources?

Fully in disagreement	Disagree	Neither agree nor disagree	Agreed	Totally agree

Question 5

Am I satisfied with the way I use technology resources?

Fully in disagreement	Disagree	Neither agree nor disagree	Agreed	Totally agree

Question 6

Do I feel I have sufficient access to literature?

Fully in disagreement	Disagree	Neither agree nor disagree	Agreed	Totally agree

Question 7

Am I satisfied with the audiobook experience?

Fully in disagreement	Disagree	Neither agree nor disagree	Agreed	Totally agree

Question 8

Do I feel that the use of the audiobook was meaningful to

Fully in disagreement	Disagree	Neither agree nor disagree	Agreed	Totally agree

Question 9

Do I enjoy the audiobook experience?

Fully in disagreement	Disagree	Neither agree nor disagree	Agreed	Totally agree

Question 10
After using the audiobook, am I interested in using other mobile applications?

Fully in disagreement	Disagree	Neither agree nor disagree	Agreed	Totally agree

Question 11

Do I feel that the audiobook experience allowed me greater access to literature?

Fully in disagreement	Disagree	Neither agree nor disagree	Agreed	Totally agree

Question 12

Do I feel that the audiobook experience makes it easier for me to have new topics of conversation?

Fully in disagreement	Disagree	Neither agree nor disagree	Agreed	Totally agree

Thank you very much for your cooperation!

Annex 3 Questionnaire to measure Usability

Select the degree of "agree" or "disagree" for each of the following items. Where:

1: Strongly disagree
2: Disagree
3: Doubtful
4: Agreed
5: Totally agree

Item	1	2	3	4	5
1.- I think I would like to use this application frequently.					
2.- I found the application unnecessarily complex.					
3.- I think the application was easy to use.					
4.- I think that I would need the support of a technical person to use this application.					
5.- I found that the functions of the application were well integrated.					
6.- I thought there was too much inconsistency in the application.					
7.- I believe that most people would learn to use the application quickly.					
8.- I found the application very complicated to use.					
9.- I felt very confident using the application.					
10.- I felt that I had to learn a lot of things before I could get going with the application.					

Annex 4

Questionnaire to measure Digital Competencies

Mark with "X" those activities that you can perform through the use of technology.

Information	Search for information on goods and services.	
	Obtaining information from authorities' websites public.	
	Read or download news/newspapers/magazines online.	
	Copy or move a file or folder.	
Communication	Send/Receive e-mails.	
	Make phone/video calls over the Internet.	
	Posting messages on chat/social networking sites.	
	Upload information to a website to be shared.	
Content creation	Use or move information in a document.	
	Use basic formulas to add, subtract, multiply or divide in a spreadsheet.	
	Create electronic presentations including, for example, images, sounds, videos, or graphics.	
	Create websites or blogs.	
	Write a computer program using a specialized programming language.	
Problem solving	Connect and install new devices to the computer.	

	Install/replace an operating system.	
	Modify or verify the configuration of a software or program.	
	Access the bank through the Internet.	
	Purchasing goods or services over the Internet.	
	Selling through the Internet.	
	Make a medical appointment through a website.	

Annex 5

Infographics for CRC dissemination

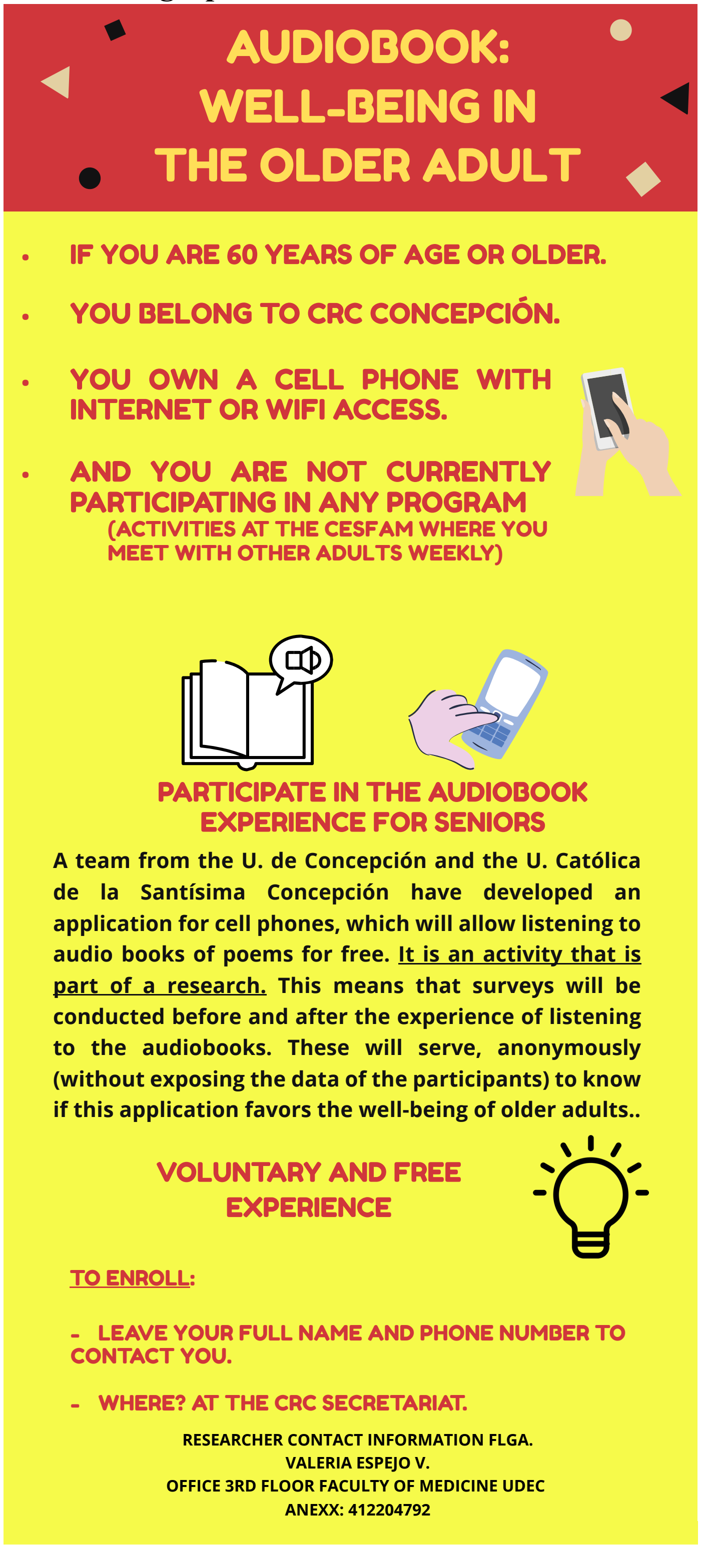

Supplement: S2 File — (PDF) [file pone.0312463.s004.pdf]
